# Development and Validation of a Deep-Learning Network for Detecting Congenital Heart Disease from Multi-View Multi-Modal Transthoracic Echocardiograms

**DOI:** 10.34133/research.0319

**Published:** 2024-03-06

**Authors:** Mingmei Cheng, Jing Wang, Xiaofeng Liu, Yanzhong Wang, Qun Wu, Fangyun Wang, Pei Li, Binbin Wang, Xin Zhang, Wanqing Xie

**Affiliations:** ^1^ Department of Intelligent Medical Engineering, School of Biomedical Engineering, Department of Psychology, School of Mental Health and Psychological Sciences, Anhui Medical University, Hefei 230011, China.; ^2^ Heart Center, Beijing Children’s Hospital, Capital Medical University, National Center for Children’s Health, Beijing 10045, China.; ^3^ School of Basic Medical Sciences, Capital Medical University, Beijing 10069, China.; ^4^ Gordon Center for Medical Imaging, Harvard Medical School, and Massachusetts General Hospital, Boston, MA 02114, USA.; ^5^ School of Life Course and Population Sciences, Faculty of Life Science and Medicine, King’s College London, London, UK.; ^6^ Center for Genetics, National Research Institute for Family Planning, Beijing 100730, China.; ^7^ Graduated School, Peking Union Medical College, Beijing 100730, China.; ^8^ Beth Israel Deaconess Medical Center, Harvard Medical School, Harvard University, Boston, MA 02215, USA.

## Abstract

Early detection and treatment of congenital heart disease (CHD) can significantly improve the prognosis of children. However, inexperienced sonographers often face difficulties in recognizing CHD through transthoracic echocardiogram (TTE) images. In this study, 2-dimensional (2D) and Doppler TTEs of children collected from 2 clinical groups from Beijing Children's Hospital between 2018 and 2022 were analyzed, including views of apical 4 chamber, subxiphoid long-axis view of 2 atria, parasternal long-axis view of the left ventricle, parasternal short-axis view of aorta, and suprasternal long-axis view. A deep learning (DL) framework was developed to identify cardiac views, integrate information from various views and modalities, visualize the high-risk region, and predict the probability of the subject being normal or having an atrial septal defect (ASD) or a ventricular septaldefect (VSD). A total of 1,932 children (1,255 healthy controls, 292 ASDs, and 385 VSDs) were collected from 2 clinical groups. For view classification, the DL model reached a mean [SD] accuracy of 0.989 [0.001]. For CHD screening, the model using both 2D and Doppler TTEs with 5 views achieved a mean [SD] area under the receiver operating characteristic curve (AUC) of 0.996 [0.000] and an accuracy of 0.994 [0.002] for within-center evaluation while reaching a mean [SD] AUC of 0.990 [0.003] and an accuracy of 0.993 [0.001] for cross-center test set. For the classification of healthy, ASD, and VSD, the model reached the mean [SD] accuracy of 0.991 [0.002] and 0.986 [0.001] for within- and cross-center evaluation, respectively. The DL models aggregating TTEs with more modalities and scanning views attained superior performance to approximate that of experienced sonographers. The incorporation of multiple views and modalities of TTEs in the model enables accurate identification of children with CHD in a noninvasive manner, suggesting the potential to enhance CHD detection performance and simplify the screening process.

## Introduction

Congenital heart disease (CHD) is typically defined as a great vessel disease at birth caused by abnormal development of the embryonic heart and vascular tissue. With a prevalence of approximately 4 to 50 per 1,000 live births, it is one of the most common congenital anomalies worldwide [1,2] and can bring a heavy health and financial burden to patients. Early CHD screening and treatment can significantly improve children’s prognosis and quality of life and prevent irreversible pulmonary vascular disease [1,3]. Among the subtypes of CHD, atrial septal defect (ASD) and ventricular septal defect (VSD) are the most common, accounting for about 10% and 40% of CHDs, respectively [4,5]. In China, the prevalence of CHD is about 0.4% to 0.7% [6]. With the advancement of medical technology and the increased dissemination of medical knowledge, CHD has gained increasing attention in China, and most simple CHDs can now be effectively treated with prompt surgery [7]. However, the medical accessibility and service quality for CHD is still low in China [8,9]. Thus, an auxiliary CHD screening system that allows inexperienced sonographers and general practitioners to perform transthoracic echocardiogram (TTE) in a simple and easy-to-use way, thereby improving the CHD screening rate and scope, is urgently needed.

Two-dimensional (2D) and Doppler ultrasound techniques reflect distinct echocardiographic information crucial for understanding cardiac structure and blood flow dynamics [10]. CHD often manifests prominent features in both 2D and Doppler TTEs, facilitating the diagnosis of CHD. However, for those inexperienced cardiac sonographers, the detection of CHD can be difficult, leading to misdiagnosis and missed diagnosis.

Deep learning (DL), one of the most advanced technologies in artificial intelligence, has been applied to various tasks in medical fields, such as disease diagnosis [11,12], lesion location [13,14], target region segmentation [15–17], abnormality detection [18], data analysis [19], measurement [20], biomedicine [21,22], and emotion recognition [23,24]. Some attempts have also tried to apply DL to the diagnosis or treatment planning for CHD [25–29]. However, developing a DL model that can comprehensively analyze multi-modal TTE images from different views to achieve more accurate identification of CHD remains a significant challenge currently under exploration.

The 2D and Doppler TTEs of 5 cardiac views from 1,932 subjects are collected in this study. Though rich information can be expected in multi-modal and multi-view depictions, it also poses challenges to exploiting the meaningful features in this high-dimensional data. In addition, some of the TTE modalities or views may be corrupted with noise or unstable motion in scanning or lost in the electronic health record transition. The regular multi-modal and multi-view TTE is often unavailable in real-world implementation while rescanning can be costly or even prohibitive. Toward the above difficulties, we integrate different TTEs to generate better diagnostic predictions through a hierarchical architecture while retaining the feasibility of flexibly handling the TTEs available. By considering more comprehensive TTE data, we hypothesized that the DL network could produce high-quality diagnostic predictions close to those of experienced clinical experts, providing richer information and better auxiliary diagnostic service.

## Results

We analyzed the TTE data of 1,308 (823 healthy controls, 209 ASDs, and 276 VSDs) and 624 children (432 healthy controls, 83 ASDs, and 109 VSDs) from the within- and cross-center datasets, respectively. More characteristics of the children in each group are summarized in Table 1. For within-center validation, the training data are randomly sampled from the within-center dataset, and the remaining data constitute the within-center test set. Instead of a fixed ratio, we used different ratios of training and testing datasets to assess the performance of the CHD detection model. To further validate the performance of our framework, the cross-center dataset is employed to test the model,s recognition results and generalization ability. The training and test data of the within-center dataset and the validation data of the cross-center dataset are randomly shuffled before being fed into the AI network.

**Table 1.** The statistics of the collected data. Data are *n*, *n* (%), or median (interquartile range).

(A) Within-center dataset				
Variable		Healthy control	ASD	VSD
2D TTE				
	A4C	823 (62.9%)	209 (16.0%)	276 (21.1%)
	SXLAX	625 (75.6%)	102 (12.3%)	100 (12.1%)
	PSLAX	820 (67.8%)	171 (14.1%)	218 (18.0%)
	PSSAX	764 (65.1%)	166 (14.2%)	243 (20.7%)
	SSLAX	641 (77.8%)	84 (10.2%)	99 (12.0%)
Doppler TTE				
	A4C	697 (71.9%)	82 (8.5%)	191 (19.7%)
	SXLAX	771 (79.2%)	92 (9.5%)	110 (11.3%)
	PSLAX	706 (73.3%)	74 (7.7%)	183 (19.0%)
	PSSAX	731 (77.3%)	68 (7.2%)	147 (15.5%)
	SSLAX	654 (81.6%)	38 (4.7%)	109 (13.6%)
Male		512 (65.2%)	83 (10.6%)	190 (24.2%)
Female		311 (59.5%)	126 (21.4%)	86 (16.4%)
Age(years)		5.5 (3.2-6.7)	2.7 (0.8-5.2)	2.2 (1.3-4.6)

(B) Cross-center dataset				
Variable		Healthy control	ASD	VSD
2D TTE				
	A4C	408 (68.0%)	83 (13.8%)	109 (18.2%)
	SXLAX	378 (76.2%)	46 (9.3%)	72 (14.5%)
	PSLAX	432 (73.6%)	67 (11.4%)	88 (15.0%)
	PSSAX	429 (80.5%)	41 (7.7%)	63 (11.8%)
	SSLAX	337 (77.3%)	33 (7.6%)	66 (15.1%)
Doppler TTE				
	A4C	376 (78.0%)	35 (7.3%)	71 (14.7%)
	SXLAX	199(76.5%)	28 (10.8%)	33 (12.7%)
	PSLAX	378 (79.7%)	34 (7.2%)	62 (13.1%)
	PSSAX	382 (78.9%)	40 (8.3%)	62 (12.8%)
	SSLAX	341 (82.8%)	29 (7.0%)	42 (10.2%)
Male		213 (76.6%)	29(10.4%)	36 (12.9%)
Female		219 (63.3%)	54 (15.6%)	73 (21.1%)
Age(years)		4.3 (2.2-7.3)	2.1 (2.1-3.7)	3.2 (0.9-4.3)

VSD, ventricular septal defect; ASD, atrial septal defect.

### Automatic view classification of TTEs

Our view classification model determines which of the 5 views (the apical 4 chambers view [A4C], the subxiphoid long-axis view [SXLAX] of 2 atria, the parasternal long-axis view [PSLAX] of the left ventricle, the parasternal short-axis view [PSSAX] of aorta, and the suprasternal long-axis view [SSLAX]) each image belongs to. When the ratio for training and testing in the first dataset is 8:2, the accuracy of the view classification model is 0.989, with a mean average precision (mAP) of 0.990 and a mean intersection over union (mIoU) of 0.979. The corresponding confusion matrix is shown in Fig. S1. With the training and test set ratio of 2:8, our view classifier can still reach an accuracy (ACC) of 0.978, an mAP of 0.978, and an mIoU of 0.957. The performance on the second evaluation dataset is 0.983 for ACC, 0.989 for mAP, and 0.973 for mIoU based on the model using 80% of data in the first dataset for training, which shows the same tendency as those in the first dataset.

To further analyze the influence of the automatic view classifier on the final CHD classification, we performed the CHD classification based on the automatic view classification results (Table S1). For the first dataset, the 5-view multi-modal CHD classifier achieved 0.992 AUC for CHD screening (normal/patient), reaching 0.984 accuracy for the classification of negative/ASD/VSD. Compared with the CHD classification results based on the view recognition identified by the clinical experts (Table S2), the computer-aided diagnostic framework with automatic view identification achieved comparable results on both within- and cross-center evaluation sets.

### CHD detection performance

#### CHD detection with multi-view TTEs

**Fig. 1.**
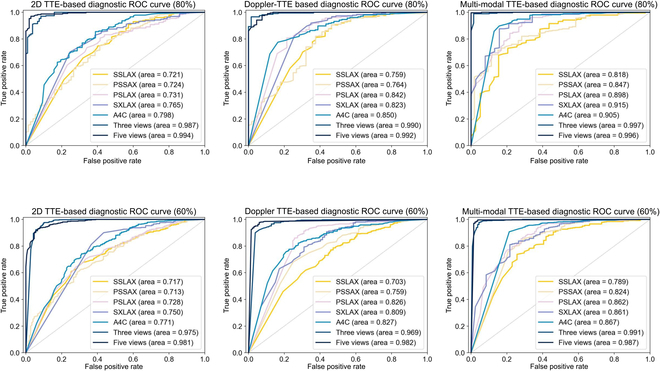
ROC curves of classification of healthy control (HC) and CHDs. ROC curves of models for CHD identification with 2D TTE (left column), Doppler TTE (middle column), and the fusion of 2D and Doppler TTEs (right column). The percentages in parentheses in the title of each figure note the proportion of training data. The top row shows the performance of models with 80% of the training data and the bottom row notes the results with 60% of the training data.

We conducted CHD identification based on each scanning view as well as the fusion of various views. For brevity, we refer to the fusion of A4C, SXLAX, and PSLAX as “3 view” for short and the fusion of 5 scanning views as “5 view” in our paper. The test results of the first dataset are shown in Table S2. When using only a single data modality, the 5-view model exhibited a 10.20% to 20.70% higher performance compared to the single-view model (training set proportion = 80%). When fusing 2 modalities of TTEs, the 5-view model exhibited a 7.00% to 12.7% higher performance than the single-view model. Furthermore, the receiver operating characteristic (ROC) curves presented in Fig. 1 provide additional evidence that the model utilizing multiple views of TTE data exhibits a significantly improved performance in recognizing CHD. The cross-center evaluation results present the consistent performance of our model (Table S2).

#### CHD detection with multi-modal TTEs

We conducted CHD detection using the 2D-TTE model, Doppler-TTE model, and multi-modal-TTE model. The prediction results are presented in Fig. 1 and Table S2. For the first dataset, when considering single-view TTEs, the multi-modal model achieved an accuracy improvement of 1.80% to 13.7% over the single-modal model (training set percentage = 80%). For 3-view and 5-view TTEs, the multi-modal model yielded higher accuracy than the single-modal model, with improvements ranging from 0.30% to 1.70% (training set percentage = 80%). The cross-center evaluation results (Table S2) also demonstrated that the fusion of modalities improves the performance of the model.

#### Comparison of CHD detection performance between the multi-view multi-modal model and human readers

**Fig. 2.**
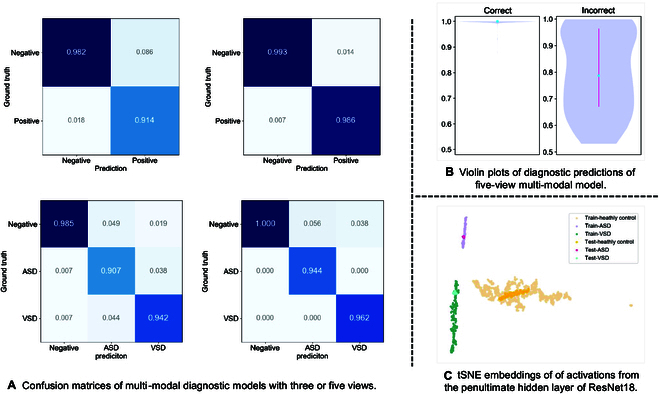
Diagnostic results of our CHD identification framework. (A) The top row denotes binary-classification confusion matrices, and the bottom row represents 3-class confusion matrices. The left column denotes the multimodal model with 3 views (A4C, SXLAX, and PSLAX) and the right column represents the multi-modal model with 5 views (A4C, SXLAX, PSLAX, PSSAX, and SSLAX). (B) Blue dots indicate the medial probability value of the corresponding predictions, and the pinkish line signifies the first to third quartiles. For the correct column, the first and third quartiles and median are all very close to 1.0. Thus, the pinkish line is not visible in the figure. (C) 2D t-distributed stochastic neighbor embedding (tSNE) embeddings of activations from the penultimate hidden layer of ResNet18. Individual points correspond to representations of various subject during training and testing (training set percentage = 80%).

One can see that the multi-view multi-modal models realized much superior discriminative performance compared to single-view or single-modal models on both test sets (Fig. 1 and Table S2). Confusion matrices of classification and violin plots of the prediction confidences of the multi-view multi-modal models are presented in Fig. 2. For the 5-view multi-modal model, the sensitivity and specificity both exceed 0.980 for distinguishing the healthy from CHD and the precision of each class are all higher than 0.940 for classifying the healthy/ASD/VSD (Fig. 2A). The mean probability of the correct predictions was almost 1.0, which was remarkably higher than that of incorrect predictions (Fig. 2B). Besides, to evaluate the feature discrimination between classes and the relationships between training and test data, we utilized 2D t-distributed stochastic neighbor embedding (tSNE) analysis (Fig. 2C and Fig. S4). Clear clustering of embeddings among healthy controls, ASDs, and VSDs was observed, along with high consistency in features between the training and testing sets. These findings provide insights into the model’s ability to distinguish between healthy controls and various subtypes of CHD.

Four primary clinicians (under 3 years of clinical experience) at Beijing Children,s Hospital also conducted CHD recognition with the same testing images. The results of primary clinicians exceed 0.910 accuracy (mean) for distinguishing the healthy from CHD and 0.860 accuracy (mean) for the classification of healthy/ASD/VSD. To further evaluate the model’s assistance for primary clinicians, these 4 doctors performed diagnosis on another 50 prospective children,s dataset with and without system support (Table 2). Whether for CHD screening or subtype determination, the accuracy of AI is much higher than that of primary doctors. With the support of the AI system, the accuracy and sensitivity of diagnosis by primary doctors have increased to about 0.95. It can be observed that our multi-view multi-modal DL framework exhibited better discriminative performance in CHD recognition compared to that of primary clinicians.

**Table 2.** Diagnostic performance of primary sonographers without and with AI support. “Bi-Accuracy” means the accuracy of the binary classification (normal/patient classifier), and “3-Accuracy” indicates the accuracy of 3-class classification (negative/ASD/VSD). “AI” denotes the CHD recognition model already trained on multi-modal TTE data with all 5 views.

Variable	Resident	Resident + AI	AI
Bi-Accuracy	0.707	0.953	0.973
Bi-Sensitivity	0.807	0.967	0.979
Bi-Specificity	0.720	0.944	0.964
3-Accuracy	0.707	0.953	0.967

### Diagnostic stability

We explored the model with training data percentages varying from 80% to 40% and conducted diagnostic classification experiments within the first dataset (Fig. 1, Tables S2 and S3). As the training ratio increased from 60% to 80%, the accuracy of the single-view single-modal models increased by 0.13% to 5.89%, while the performance of the multi-view and multi-modal models was boosted by 0.61% to 1.34%. As the volume of training data decreases, the diagnostic performance also decreases, particularly for the single-view single-modal models. However, the impact of decreased training data on multi-view and multi-modal models is significantly less pronounced. When the training set proportion drops to 40%, the 5-view multi-modal model can still perform effectively with an AUC of 0.983 for normal/patient classification and an accuracy of 0.968 for negative/ASD/VSD classification.

To investigate the recognition stability of the CHD diagnostic framework based on different DL models, we employed AlexNet [30], Vgg13 [31], and ResNet50 [32] for CHD detection. Descriptions of these network structures can be found in Fig. S2 and Appendix S6, and the corresponding results and analysis are shown in Table S4 and Appendix S7.

### High-risk region visualization

**Fig. 3.**
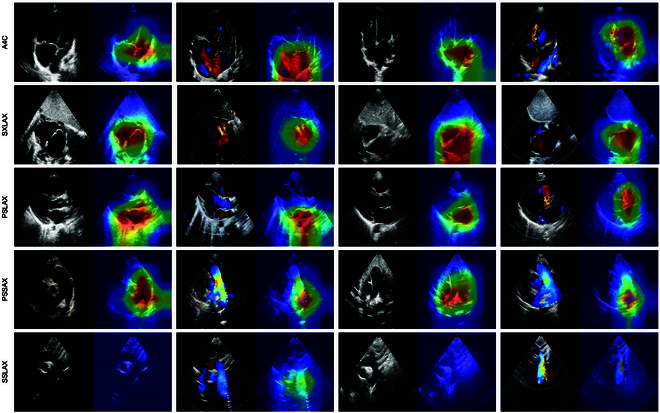
CAM figures of testing images. The first 2 columns indicate ASD, and the last 2 columns signify VSD. The red area shows the location to which the model pays the most attention.

By employing the gradient-weighted class activation mapping approach [33], we can visualize the regions in the images that are most relevant for distinguishing ASD and VSD in the DL models (Fig. 3). For instance, in the A4C view of 2D TTE, the model,s attention to ASD was focused on the location of the defect of the atrial septum, the right atrium with an enlarged inner diameter, and the left atrium with a reduced inner diameter. In the PSLAX view of Doppler TTE, the model,s identification of VSD was focused on the location of the defect and its left-to-right shunt signal, and the left atrium with an enlarged inner diameter. More visual results and detailed analysis are presented in Fig. S3 and Appendix S8. It is evident that the regions of interest identified by the DL model exhibited strong similarities to those identified by clinicians.

## Discussion

Early screening and intervention for CHD are of significant importance [34]. However, due to the complexity of CHD, the limitations of screening technology, and the limited experience of doctors in some underdeveloped areas, diagnosis of CHD remains a great challenge in many regions of the world. Although there is a great demand for an accurate, flexible, and stable CHD recognition system, developing an effective AI auxiliary diagnostic model that integrates multiple types of TTEs to attain the diagnostic proficiency of an experienced clinician remains a challenge. In this study, we collected 2D and Doppler TTEs with 5 views from 1,932 children, providing a new opportunity to investigate the performance of a DL recognition model in identifying pathology patterns across different views and modalities.

Our comprehensive multi-view multi-modal CHD recognition model showed superior performance in CHD screening on both within- and cross-center test sets. Our model reached an AUC of 0.996 and an accuracy of 0.991 for negative/ASD/VSD classification (using a training data proportion of 80% on the first dataset), which approximates the diagnostic accuracy of experienced sonographers. This performance demonstrated that the framework has the potential to assist not only in the early and large-scale screening for CHD but also in clinical diagnosis and may aid in the discrimination of more subtypes. Furthermore, the multi-view multi-modal model exhibited significantly higher diagnostic performance than the single-view single-modal model. This demonstrated that the diagnostic model based on deep-learning technology not only can efficiently process a large amount of data, but also can fully mine and analyze the information in multiple TTE images, so as to reach the diagnostic proficiency of experienced clinicians. As a systematic detection system, our model extracts images from each view and modality separately, and then generates prediction information through feature fusion. Therefore, when only single-modal or single-view TTE data are available, our model can also provide clinical diagnostic information, such as prediction probability and high-risk region saliency map. This reveals the framework,s flexible detection capability to adapt to different clinical scenarios. For instance, even in the event of partial loss of TTE data during storage and transmission, our system is still capable of diagnostic prediction, which provides great convenience for clinical data collection and computer-aided CHD screening.

The framework can also be used as standalone software to analyze data for studies, as well as online software to perform real-time detection of CHD, benefiting from its high performance and quick inference speed (lower than 2.3 ms for CHD classification per subject). Based on the keyframe selection scheme, the multi-view multi-modal CHD classification model not only obtains effective information for detection but also reduces the computational speed and memory space occupation, thereby reducing the system’s requirements for hardware devices and improving the practicality and scope of clinical application. Furthermore, the excellent implantability and scalability of our model allow it to be embedded in many clinical devices and process diverse data, thereby facilitating the implementation of telemedicine.

Generally, in most studies, the test set proportion ranges from 10% to 20%. However, in this study, we used various test set ratios ranging from 20% to 60% to observe the stability of the model. When the test set size was 1.5 times that of the training set, our multi-view multi-modal recognition framework still achieved superior results, with an AUC of 0.983 for screening and an ACC of 0.968 for classifying negative/ASD/VSD. In addition, there are many reasons that can lead to low-quality TTEs in real-life clinical environments. Therefore, an AI model trained with manually selected high-quality data cannot offer reliable prediction facing real-life data. The dataset in this study was all collected from real clinical settings, and the isovolumic relaxation phase TTEs were obtained through an automatic keyframe selection rather than a manual selection. The multi-view and multi-modal model architecture greatly enhances the model’s resistance to noise and interference in the data, improves the model’s robustness, and allows it to be performed more reliably in clinical applications.

The number of images in each 2D and Doppler study can already reach as many as thousands, not to mention the huge amount of video data of 2 modalities from 5 views. It is necessary to free observers from the repetitive work of manually identifying the keyframe TTE for diagnosis and distinguishing different views of images, which is very time-consuming. The experimental results demonstrate the superior performance of our scanning view discriminability and simultaneously illustrate how our overall framework for CHD detection can serve clinical diagnosis in multiple aspects. By fusing the view classifier with CHD detection into a systematic framework, it provides support for computer-aided recognition systems to adaptively fuse various types of TTE data for more flexible and accurate diagnosis, as well as further promote the generalization for the screening of other subtypes of CHD and even other rare heart diseases.

The CAM figures showed the region in images that contributed the most to diagnostic prediction, giving a deep insight into the inference scheme of the DL model, which highly correlated with the structural information of the heart that is of clinical concern in CHD. Considering the complexities of CHD and clinical courses, it is crucial to identify and visually represent regions that signify high-risk information, providing more detailed information on the location of interest in each TTE image and facilitating subsequent surgical planning.

Our study has several limitations. ASD and VSD are the most common subtypes of CHD; it is promising to collect more data subtypes for a more comprehensive detection of CHD. In addition, TTE is widely used in clinical assessment of cardiac-related symptoms. Thus, other rare heart diseases can be incorporated into our study, providing convenience for early screening of more heart diseases. Besides, more diverse and detailed information needs to be explored, such as target location, distance, and area size, so that the system can provide more comprehensive information to serve clinical needs, especially by offering better support for the surgical plan of CHD, which is the direction of our future work. In addition, data from other vendors of ultrasound equipment need to be considered, which is the future work that we will keep working on.

Our study has developed and validated a comprehensive framework for CHD detection using 2D and Doppler TTE images from various scanning views, including A4C, SXLAX, PSLAX, PSSAX, and SSLAX. The system exhibited excellent performance in classifying scanning views and identifying healthy individuals, as well as those with ASD or VSD. The results suggest that the model has the potential to facilitate and improve widespread screening and the distinguishing of CHD subtypes in children.

## Materials and Methods

### Study design and data collection

This study utilized 2D and Doppler TTE images of 1,932 children (1,255 healthy controls, 292 ASDs, and 385 VSDs) from 2 clinical groups from Beijing Children’s Hospital between 2018 and 2022. The first dataset contained 1,308 children (823 healthy controls, 209 ASDs, and 276 VSDs), and the second dataset included 624 children (432 healthy controls, 83 ASDs, and 109 VSDs) collected from different sonographers in Beijing Children’s Hospital. The study protocol was approved by the Ethics Committee of Beijing Children’s Hospital (No. 2019-k-342), and all samples recruited in the present study were from outpatients or inpatients of the heart center. After the TTE examination, the TTE data from a total of 1,932 individuals with no cardiac structural abnormalities were used as healthy control data. The individuals diagnosed with ASD or VSD were recruited as positive cases. All subjects were determined by at least 2 experienced senior sonographers (chief physician, over 15 years of experience, over 150,000 ultrasound examinations) or intraoperative final diagnosis. Among CHD patients, we also recorded the following treatment: 196 ASD and 70 VSD patients were treated with transcatheter closure, while 96 ASD and 315 VSD patients were treated with surgical closure. More detailed statistics of the within- or cross-center dataset are shown in Table 1.

PHILIPS iE 33, iE Elite, and EPIQ 7C (Philips Electronics Nederland B.V.) with transducer frequency ranging from 3 to 8 MHz were adopted as data acquisition equipment. Given the analysis of the atrial septum and ventricular septum, the heart defect was identified, and cavity or pulmonary venous return was noted [19]. Each patient was placed in the supine position and captured with 1 to 5 views of 2D and Doppler-TTE data, providing sufficient information for clinical AI-assisted detection. According to the recommendation of clinical experts, we collected 5 standard views: A4C, SXLAX, PSLAX, PSSAX, and SSLAX, which provide sufficient information for the diagnosis of most CHDs. To facilitate the TTE data collection, processing, and analysis, we adopted a keyframe selection method [5], which selects the isovolumic relaxation phase as the keyframe when the ventricles finish contracting and start to relax. More description of data collection, data pre-processing, and the strategies for handling class imbalance can be found in Appendix S1, Appendix S2, and Fig. S5.

### Framework development

**Fig. 4.**
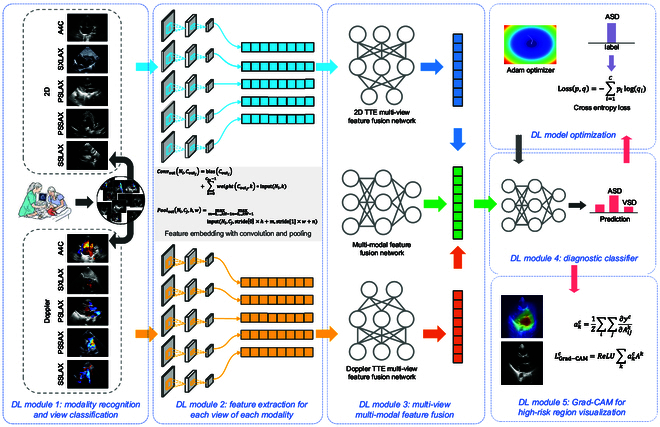
Overview of the CHD detection framework. The CHD detection model first identifies the modality and view of the TTE images. Then, the model extracts features from each view of each modality. After fusing the multi-view multi-modal features, the model generates predictions of negative, ASD, or VSD and visualizes the high-risk regions that are most related to CHD.

The overall CHD detection framework consists of scanning view and modality recognition, CHD diagnostic prediction, and high-risk region visualization. The diagnostic part first explores the information from every 5 scanning images of 2 modalities with 10 sub-models. It then comprehensively investigates the coupling relationships in the high-dimensional embedding space of hidden layers of the DL model to provide the diagnostic classifier with more reliable features. This hierarchical architecture helps generate more accurate probability predictions of whether each subject is normal, has ASD, or has VSD. The overview of our framework is shown in Fig. 4.

Concretely, the distinction between 2D and Doppler TTE can be directly realized through the color channel measurement. For view classification, the classic DL network ResNet18 [23] was adopted to recognize which scanning view the image belongs to. To detect CHD using TTEs, we constructed a feature extraction module for each modality with a particular view using ResNet18. We also built a multi-level feature fusion network with a fully connected neural network (FCNN) to fuse the features. Finally, we employed a small FCNN module to construct the diagnostic classifier, which computes the probability of each subject being normal, having ASD, or having VSD. More details of the models, technical settings, and the training and inference time can be found in Appendix S3 and Appendix S4.

### Statistical analysis

The ACC and area under the receiver operating characteristic curve (AUC) were chosen as the primary outcomes for CHD screening. We also employed the metrics of precision, sensitivity, and specificity, which provided further insights into the model,s ability to detect positive and negative samples. To assess the performance of our DL model in identifying the subtypes of CHD, we adopted ACC as the primary outcome. Additionally, we calculated the confusion matrix, mAP, and the mIoU to gain detailed information on the classification of each subtype and the model,s subtype recognition performance. Cross-center test was further conducted to evaluate the generality of our CHD identification framework, and the same metrics as those of within-center evaluation were adopted for the cross-center evaluation. All experiments were implemented 10 times to compute the standard deviation.

In general, to train and test the performance of a model, the dataset is partitioned into training and testing sets at a fixed ratio, such as 8:2. However, for many AI models, the size of the training dataset has a significant impact on the model,s detection ability, which may sharply decline as the proportion of the training set decreases. To evaluate the stability of our CHD detection model under different training set ratios, we trained and tested our CHD recognition framework using the within-center dataset with various training set proportions ranging from 80% to 40% and the remaining data for testing. We used the ACC and AUC for CHD screening and the ACC for CHD subtype discrimination to observe whether the CHD recognition framework can maintain effective and stable detection results with a reduced amount of training data. Furthermore, we used the same metrics to investigate the influence of different DL architectures on the performance of the CHD detection framework using multi-view and multi-modal TTEs.

The performance of scanning view distinguishing was evaluated using ACC, mAP, mIoU, and the confusion matrix. More details of the statistical analysis are described in Appendix S5.

## Data Availability

All data collected for the study are not publicly available for download regarding patient confidentiality and consent. However, the corresponding authors can be contacted for academic inquiry.

## Supplementary Materials

Supplementary 1 Appendices S1 to S8 Figs. S1 to S5 Tables S1 to S5

## References

[1] Zhao L, Chen L, Yang T, Wang T, Zhang S, Chen L, Ye Z, Luo L, Qin J. Birth prevalence of congenital heart disease in China, 1980–2019: A systematic review and meta-analysis of 617 studies. Eur J Epidemiol. 2020;35(7):631–642. 32519018 10.1007/s10654-020-00653-0 PMC7387380

[2] Lin C, Liu L, Liu Y, Leng J. Recent developments in next-generation occlusion devices. Acta Biomater. 2021;128:100–119. 33964482 10.1016/j.actbio.2021.04.050

[3] Zhao Q, Ma X, Ge X, Liu F, Yan WL, Wu L, Ye M, Liang XC, Zhang J, Gao Y, et al. Pulse oximetry with clinical assessment to screen for congenital heart disease in neonates in China: A prospective study. Lancet. 2014;384(9945):747–754. 24768155 10.1016/S0140-6736(14)60198-7

[4] Geva T, Martins JD, Wald RM. Atrial septal defects. Lancet. 2014;383(9932):1921–1932. 24725467 10.1016/S0140-6736(13)62145-5

[5] Hoffman JI, Kaplan S, Liberthson RR. Prevalence of congenital heart disease. Am Heart J. 2004;147(3):425–439. 14999190 10.1016/j.ahj.2003.05.003

[6] Wu L, Li B, Xia J, Ji C, Liang Z, Ma Y, Li S, Wu Y, Wang Y, Zhao Q. Prevalence of congenital heart defect in Guangdong province, 2008-2012. BMC Public Health. 2014;14:152. 24517105 10.1186/1471-2458-14-152 PMC3928880

[7] Wu K, Lü X, Liu Y. Recent progress of pediatric cardiac surgery in China. Chin Med J. 2006;119(23):2005–2012. 17199946

[8] Fullman N, Yearwood J, Abay SM, Abbafati C, Abd-Allah F, Abdela J, Abdelalim A, Abebe Z, Abebo TA, Aboyans V, et al. Measuring performance on the healthcare access and quality index for 195 countries and territories and selected subnational locations: A systematic analysis from the global burden of disease study 2016. Lancet. 2018;391(10136):2236–2271. 29893224 10.1016/S0140-6736(18)30994-2 PMC5986687

[9] Pang L, Lu J, Song H. Impact of delayed diagnosis time on post-surgery recovery in 0–18 years old congenital heart disease patients in 8 western provinces of China: A comparative analysis. Chin J Public Health. 2022;38(6):671–675.

[10] Mcleod G, Shum K, Gupta T, Chakravorty S, Kachur S, Bienvenu L, White M, Shah SB. Echocardiography in congenital heart disease. Prog Cardiovasc Dis. 2018;61:468–475. 30445162 10.1016/j.pcad.2018.11.004

[11] Klang E, Grinman A, Soffer S, Margalit Yehuda R, Barzilay O, Amitai MM, Konen E, Ben-Horin S, Eliakim R, Barash Y, et al. Automated detection of Crohn’s disease intestinal strictures on capsule endoscopy images using deep neural networks. J Crohn’s Colitis. 2021;15(5):749–756. 33216853 10.1093/ecco-jcc/jjaa234

[12] Elias P, Poterucha TJ, Rajaram V, Moller LM, Rodriguez V, Bhave S, Hahn RT, Tison G, Abreau SA, Barrios J, et al. Deep learning electrocardiographic analysis for detection of left-sided valvular heart disease. J Am Coll Cardiol. 2022;80(6):613–626. 35926935 10.1016/j.jacc.2022.05.029

[13] Nguyen EH, Yang H, Deng R, Lu Y, Zhu Z, Roland JT, Lu L, Landman BA, Fogo AB, Huo Y. Circle representation for medical object detection. IEEE Trans Med Imaging. 2021;41(3):746–754. 10.1109/TMI.2021.3122835 PMC8963364 34699352

[14] Kaur A, Singh Y, Neeru N, Kaur L, Singh A. A survey on deep learning approaches to medical images and a systematic look up into real-time object detection. Arch Comput Methods Eng. 2022;29:2071–2111.

[15] Bian X, Luo X, Wang C, Liu W, Lin X. DDA-net: Unsupervised cross-modality medical image segmentation via dual domain adaptation. Comput Methods Prog Biomed. 2022;213: Article 106531. 10.1016/j.cmpb.2021.106531 34818619

[16] Guo H, Yang D. PRDNet: Medical image segmentation based on parallel residual and dilated network. Measurement. 2021;173: Article 108661.

[17] Singh G, Alaref S, Maliakal G, Pandey M, van Rosendael A, Lee B, Wang J, Xu Z, Min J. Deep learning based automatic segmentation of cardiac computed tomography. J Am Coll Cardiol. 2019;73:1643–1643.30947916

[18] Kusunose K, Abe T, Haga A, Fukuda D, Yamada H, Harada M, Sata M. A deep learning approach for assessment of regional wall motion abnormality from echocardiographic images. JACC Cardiovasc Imaging. 2020;13:374–381. 31103590 10.1016/j.jcmg.2019.02.024

[19] Rodrigo M, Rogers A, Ganesan P, Krittanawong C, Alhusseini M, Narayan S. Classification of individual atrial intracardiac electrograms by deep learning. J Am Coll Cardiol. 2021;77:3217–3217.34167646

[20] Salte IM, Østvik A, Smistad E, Melichova D, Nguyen TM, Karlsen S, Brunvand H, Haugaa KH, Edvardsen T, Lovstakken L, et al. Artificial intelligence for automatic measurement of left ventricular strain in echocardiography. JACC Cardiovasc Imaging. 2021;14(10):1918–1928. 34147442 10.1016/j.jcmg.2021.04.018

[21] Li J, Chen J, Bai H, Wang H, Hao S, Ding Y, Peng B, Zhang J, Li L, Huang W. An overview of organs-on-chips based on deep learning. Research. 2022;2022:9869518. 35136860 10.34133/2022/9869518 PMC8795883

[22] Qi R, Zou Q. Trends and potential of machine learning and deep learning in drug study at single-cell level. Research. 2023;6:0050. 36930772 10.34133/research.0050 PMC10013796

[23] Fan C, Yao L, Zhang J, Zhen Z, Wu X. Advanced reinforcement learning and its connections with brain neuroscience. Research. 2023;6:0064. 36939448 10.34133/research.0064 PMC10017102

[24] Zhou Y, Ren F. CERG: Chinese emotional response generator with retrieval method. Research. 2020. 10.34133/2020/2616410 PMC7510341 33015633

[25] Wang J, Liu X, Wang F, Zheng L, Gao F, Zhang H, Zhang X, Xie W, Wang B. Automated interpretation of congenital heart disease from multi-view echocardiograms. Med Image Anal. 2021;69: Article 101942. 33418465 10.1016/j.media.2020.101942

[26] Arnaout R, Curran L, Zhao Y, Levine JC, Chinn E, Moon-Grady A. An ensemble of neural networks provides expert-level prenatal detection of complex congenital heart disease. Nat Med. 2021;27(5):882–891. 33990806 10.1038/s41591-021-01342-5 PMC8380434

[27] Arnaout R. Toward a clearer picture of health. Nat Med. 2019;25(1):12. 30613101 10.1038/s41591-018-0318-x

[28] Ouyang D, He B, Ghorbani A, Yuan N, Ebinger J, Langlotz CP, Heidenreich PA, Harrington RA, Liang DH, Ashley EA, et al. Video-based AI for beat-to-beat assessment of cardiac function. Nature. 2020;580:252–256. 32269341 10.1038/s41586-020-2145-8 PMC8979576

[29] Wang J, Xie W, Cheng M, Wu Q, Wang F, Li P, Fan B, Zhang X, Wang B, Liu X. Assessment of transcatheter or surgical closure of atrial septal defect using interpretable deep keypoint stadiometry. Research. 2022;2022:9790653. 36340508 10.34133/2022/9790653 PMC9620637

[30] Krizhevsky A, Sutskever I, Hinton GE. ImageNet classification with deep convolutional neural networks. Commun ACM. 2017;60(6):84–90.

[31] Simonyan K, Zisserman A. Very deep convolutional networks for large-scale image recognition. arXiv. 2015. https://arxiv.org/abs/1409.1556

[32] He K, Zhang X, Ren S, Sun J. Deep residual learning for image recognition. Paper presented at: 2016 IEEE Conference on Computer Vision and Pattern Recognition (CVPR); 2016 Jun 27–30; Las Vegas, NV.

[33] Selvaraju RR, Cogswell M, Das A, Vedantam R, Parikh D. Batra D Grad-CAM: Visual explanations from deep networks via gradient-based localization. Paper presented at: Proceedings of the IEEE International Conference on Computer Vision; 2017 Oct 22–29; Venice, Italy.

[34] Hoskoppal A, Roberts H, Kugler J, Duncan K, Needelman H. Neurodevelopmental outcomes in infants after surgery for congenital heart disease: A comparison of single-ventricle vs. two-ventricle physiology. Congenit Heart Dis. 2010;5(2):90–95. 20412480 10.1111/j.1747-0803.2009.00373.x

