# Supplementary material for: Development and Validation of a Deep-Learning Network for Detecting Congenital Heart Disease from Multi-View Multi-Modal Transthoracic Echocardiograms
Source: Research (Wash D C). 2024 Mar 6;7:0319. doi: 10.34133/research.0319 (PMC10919123; doi:10.34133/research.0319)
Supplement: Supplementary 1 — Appendices S1 to S8 Figs. S1 to S5 Tables S1 to S5 [file research.0319.f1.zip › efigure1.pdf]

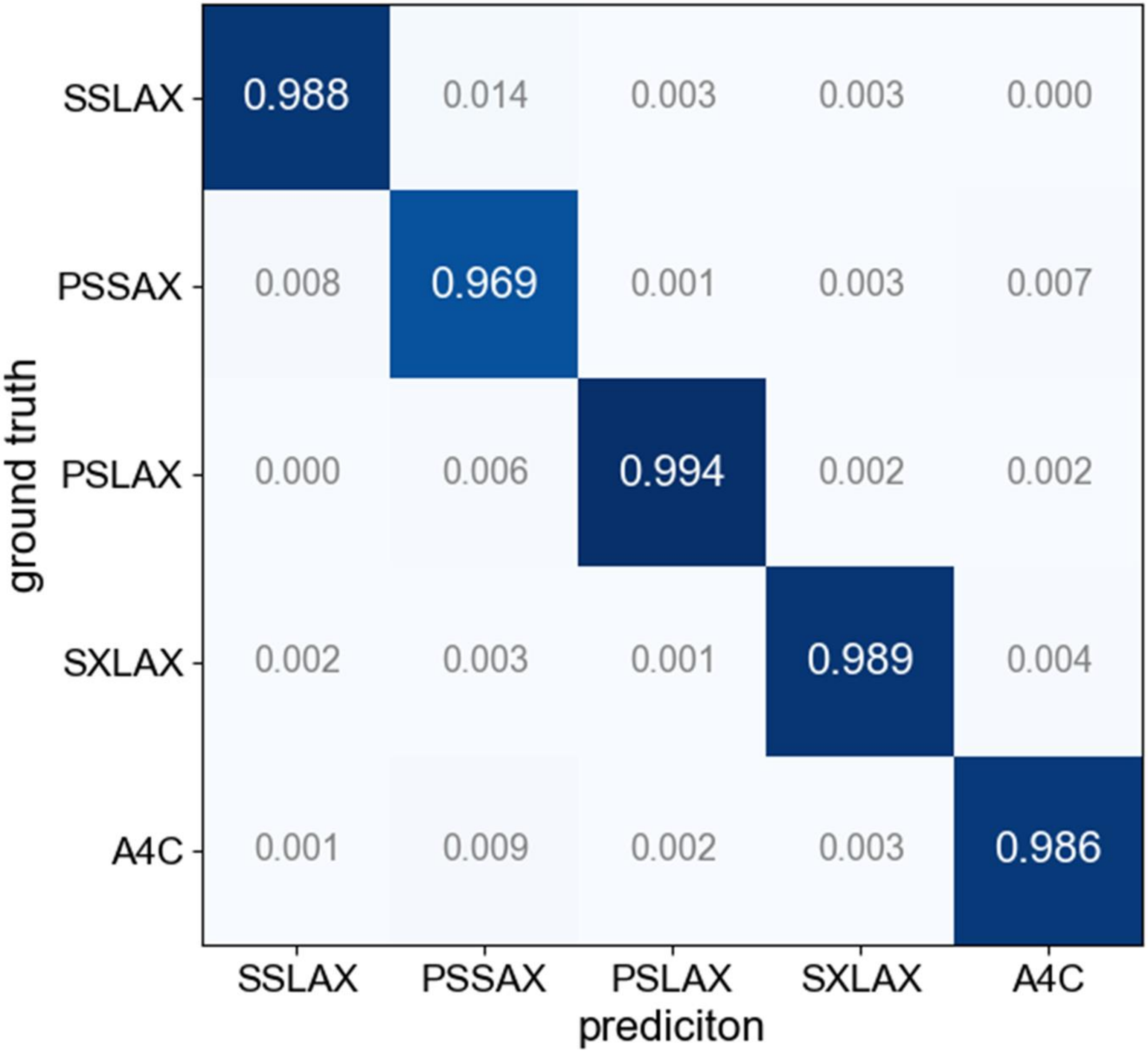

eFigure 1. The confusion matrix of view classification with the training data ratio of 80% for the within-center evaluation.
