# Supplementary material for: Development and Validation of a Deep-Learning Network for Detecting Congenital Heart Disease from Multi-View Multi-Modal Transthoracic Echocardiograms
Source: Research (Wash D C). 2024 Mar 6;7:0319. doi: 10.34133/research.0319 (PMC10919123; doi:10.34133/research.0319)
Supplement: Supplementary 1 — Appendices S1 to S8 Figs. S1 to S5 Tables S1 to S5 [file research.0319.f1.zip › efigure3.pdf]

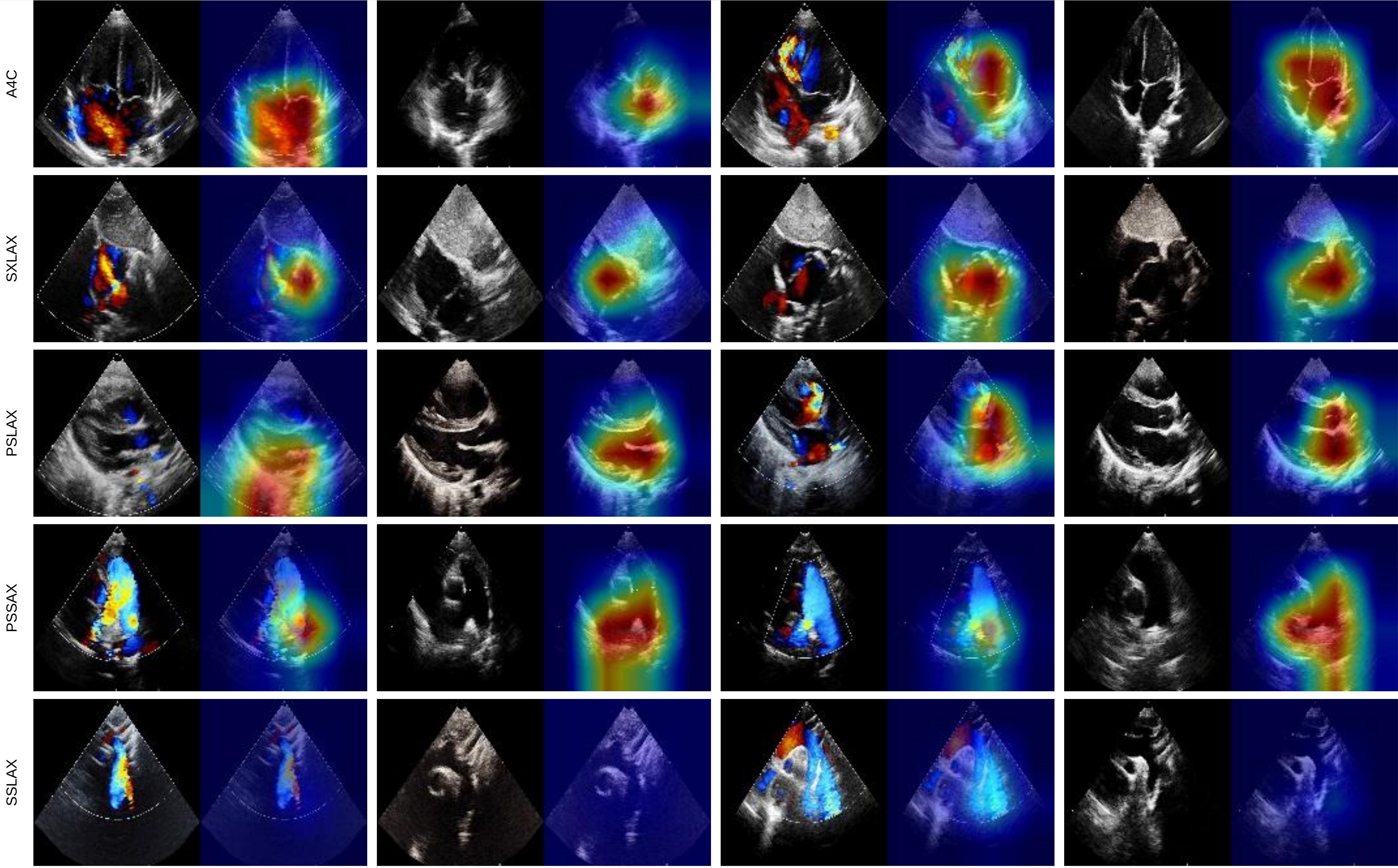

eFigure 3. Testing images with corresponding CAMs. The left two columns indicate ASD, and the right two columns signify VSD. The red area shows the location to which the model pays the most attention.
