# Supplementary material for: Development and Validation of a Deep-Learning Network for Detecting Congenital Heart Disease from Multi-View Multi-Modal Transthoracic Echocardiograms
Source: Research (Wash D C). 2024 Mar 6;7:0319. doi: 10.34133/research.0319 (PMC10919123; doi:10.34133/research.0319)
Supplement: Supplementary 1 — Appendices S1 to S8 Figs. S1 to S5 Tables S1 to S5 [file research.0319.f1.zip › efigure4.pdf]

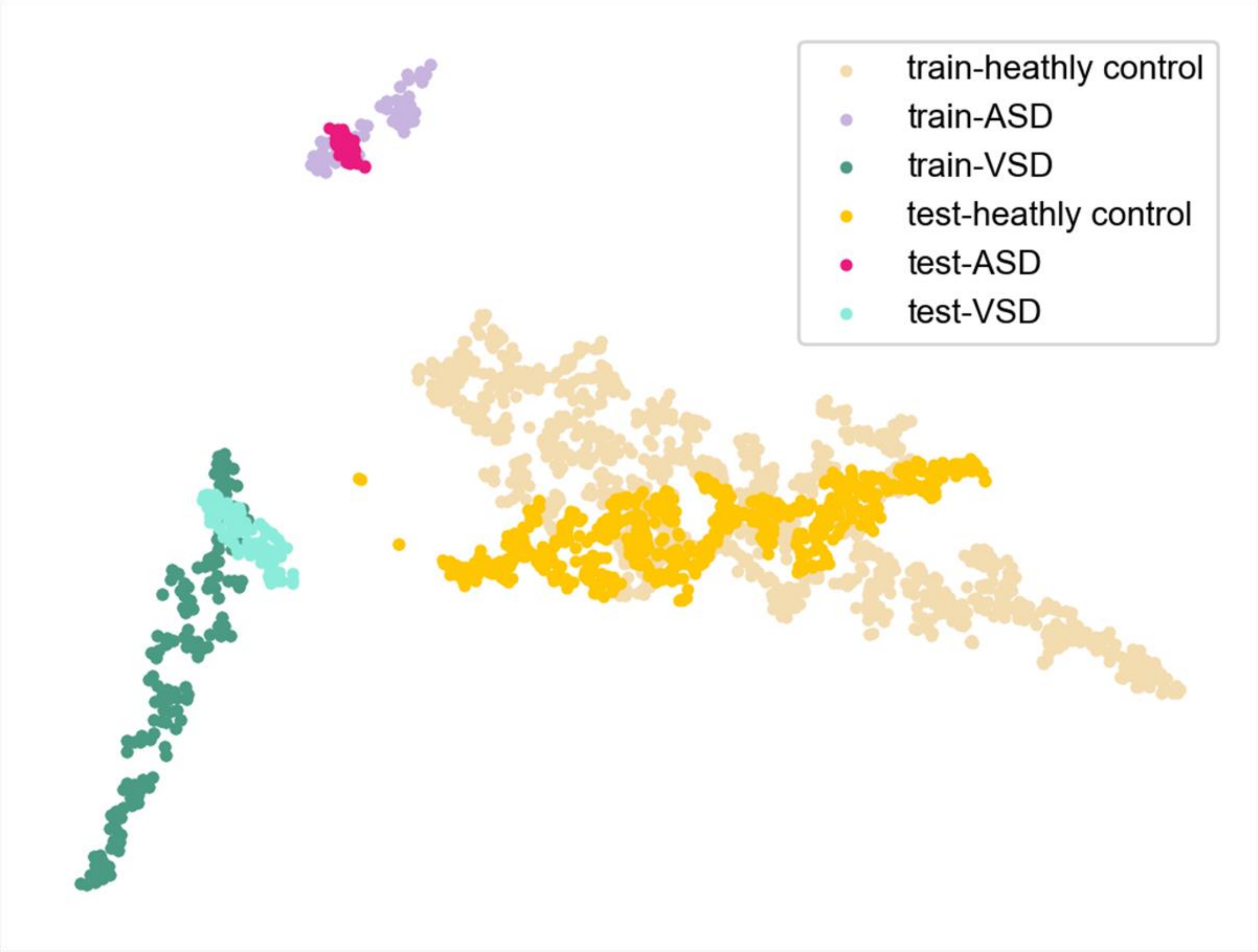

eFigure 4. Feature observation of the DL network. Two-dimensional (2D) t-distributed stochastic neighbor embedding (tSNE) of activations from the penultimate hidden layer of ResNet18 for the first dataset. Individual points correspond to representations of various subjects during training and testing (training set percentage=60%).
