# Supplementary material for: Development and Validation of a Deep-Learning Network for Detecting Congenital Heart Disease from Multi-View Multi-Modal Transthoracic Echocardiograms
Source: Research (Wash D C). 2024 Mar 6;7:0319. doi: 10.34133/research.0319 (PMC10919123; doi:10.34133/research.0319)
Supplement: Supplementary 1 — Appendices S1 to S8 Figs. S1 to S5 Tables S1 to S5 [file research.0319.f1.zip › eFigure5.pdf]

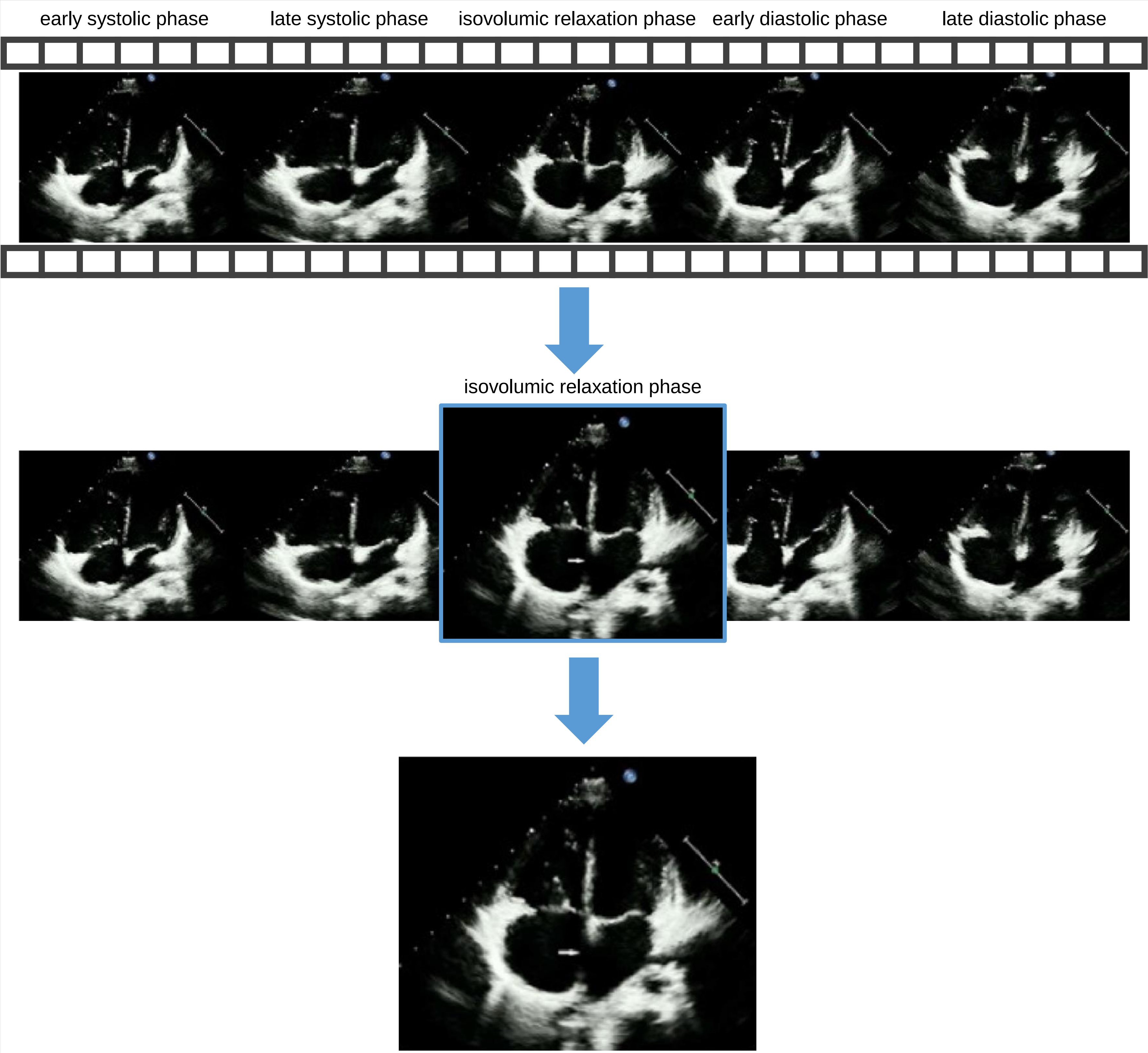


**eFigure 5 Keyframe selection flowchart.** The flowchart of keyframe selection from the A4C TTE video of an ASD patient.
