# Supplementary material for: Development and Validation of a Deep-Learning Network for Detecting Congenital Heart Disease from Multi-View Multi-Modal Transthoracic Echocardiograms
Source: Research (Wash D C). 2024 Mar 6;7:0319. doi: 10.34133/research.0319 (PMC10919123; doi:10.34133/research.0319)
Supplement: Supplementary 1 — Appendices S1 to S8 Figs. S1 to S5 Tables S1 to S5 [file research.0319.f1.zip › revised_supplementary_20240116.docx]

**Development and validation of deep-learning network for detecting congenital heart disease from multi-view multi-modal transthoracic echocardiograms**

**Supplementary Material**

**eAppendix 1.** Data collection

**eAppendix 2.** Data pre-processing

**eAppendix 3.** Framework development

**eAppendix 4.** Training and testing settings

**eAppendix 5.** Statistical Analysis

**eAppendix 6.** Structure of DL models

**eAppendix 7.** Diagnostic stability with more DL networks

**eAppendix 8.** High-risk region visualziation

**eFigure 1.** The confusion matrix of view classification with the training data ratio of 80%

**eFigure 2.** The DL network structures for adopted in our framework

**eFigure 3.** Testing images with corresponding CAMs

**eFigure 4.** More feature observation of the DL network

**eFigure 5.** Keyframe selection flowchart

**eTable 1.** The CHD diagnostic results based on the automatic view identification

**eTable 2.** CHD recognition results of our multi-modal CHD identification model with various scanning views

**eTable 3.** Quantitative diagnostic results based on different modalities and scanning views with 50% and 40% training ratios

**eTable 4.** Results of diagnostic model based on different DL networks

**eTable 5.** Diagnostic performance of primary sonographers without and with AI support

**eAppendix 1. Data collection**

In this study, we collected 1,932 patients (1,255 healthy controls, 292 ASDs, and 385 VSDs, 1063 males and 869 females, ages ranging from 9 months to 7 years 3 months) from the outpatients or inpatients of the heart center in Beijing Children's Hospital (BCH) between 2018 and 2022. Each child was determined by at least two experienced senior sonographers or intraoperative final diagnosis. After transthoracic echocardiogram (TTE) examination, the individuals with no cardiac structural abnormalities are enrolled in the control group, and the children with atrial septal defect (ASD) or ventricular septal defect (VSD) are included in the positive cohort.

It is worth noting that only the TTE data at the first diagnosis were used. In China, due to the shortage of pediatricians and sonographers, during the first examination of children, sonographers perform rapid screening for CHD based on TTE examination. When further observation and measurement are needed to achieve CHD precise subtype classification and surgical plan formulation, a more detailed TTE examination will be performed. In order to better adapt to clinical practice, we did not deliberately select or specifically collect data that can perfectly display the defect but only collected the TTE data with the five views at the first diagnosis.

In order to reduce mistakes during TTE examinations, we adopted a standard operating procedure (SOP) with a step-by-step and repeatable process of transthoracic echocardiogram (TTE) examination and provided standard operation training for all sonographers. It improves the plane and view selection of TTE and enhances the consistency and standardization of TTE images. Although the standardization and consistency of collected TTE images can be basically enhanced by using the SOP of TTE examination, there are many reasons that can lead to the degradation of image quality in real-life clinical environments. Thus, a robust and reliable CHD recognition system that can resist noise and interference in data is very necessary to help clinical practice.

The original collected data of each view from the clinical is an echocardiogram video with three cardiac cycles. To reduce redundant data, we randomly crop a clip of 0.8 seconds from each video since the cardiac cycle of the child is typically about 0.5–0.6s. To further facilitate the data processing, we then employed an automatic keyframe selection method(1) on each cropped video. We adopted the isovolumic relaxation phase as the keyframe when the ventricles finish contracting and start to relax, while the defects of VSD and ASD can be shown clearly at that time.

Specifically, we used a temporal convolution module to efficiently explore the spatial-temporal cues to assign the weight to each frame in an echocardiogram video. The training was supervised by the keyframe label in the training dataset, which was manually annotated by the experienced ultrasound doctors. Then, the frame with the highest weight was employed as the keyframe. Based on the keyframe selection algorithm, the CHD auxiliary diagnosis system can obtain the representative keyframe information from the video while greatly reducing the computational complexity of the AI model and avoiding the high computing time and memory costs caused by the video.

Overall, the data collection process can be summarized as follows: First, we conducted standard TTE examination training for each sonographer from the two clinical teams of Beijing Children's Hospital. Among outpatients and inpatients at the heart center, the data of CHD patients and children with no cardiac structural abnormalities were selected, each of whom was determined by at least two experienced senior sonographers or intraoperative final diagnosis. Then, 2D and Doppler TTE videos from 5 standard cardiac views of each selected individual were collected. Based on the keyframe automatic selection method(1), we obtain the keyframe in each video and construct the within- and cross-center datasets. For the within-center dataset, the training and test sets are randomly selected according to a certain proportion. All data in the cross-center dataset are used for external validation and sent to the model one by one for testing after the random shuffle.

The diagnosis of different subtypes of CHD relies differently on distinct views, but the five views of 2D and Doppler TTE collected in our study can already provide sufficient information for the screening of most CHDs. However, compared with ASD and VSD, the incidence of other CHD subtypes is relatively lower, so the data on these subtypes is still being collected, such as patent ductus arteriosus (PDA), tetralogy of Fallot (TOF), and patent foramen ovale (PFO), and we will continue to advance research on other subtypes.

**eAppendix 2. Data pre-processing**

Some instrument marks often exist in the collected clinical raw echocardiogram images. In our dataset, only positive samples (patients) have electrocardiogram records. To prevent the network from being lazy in producing predictions through these clues, we need to erase these extra marks for fair experiments. First, we cropped the raw image and kept the region of interest (ROI) to remove the surrounding irrelevant areas. Then, given the sector structure of the ultrasonic image, we filtered the ROI image with a sector mask. In this way, we can extract the ultrasonic region we need and remove the redundant parts. Next, we resized all masked ROI images to 128×128 to normalize the size of the network input.

In our dataset, the number of negative samples (healthy control) is much more than that of positive samples for CHD classification. The wide source healthy group makes this phenomenon very common, but it also causes the predictions of the network to be potentially more inclined to the negative. To this end, a data augmentation strategy on the training set was adopted to deal with the class-imbalanced problem. Specifically, for those positive training samples that do not have all five views of both two modalities, we randomly sample the missed view of the modality from other negative training samples to compose "virtual" patients. There are two reasons why images of the negative were chosen to construct the "virtual" positive sample rather than the positive data of the same class. On the one hand, the initially available data can already provide sufficient information for clinical diagnosis, and using negative sample data to supplement missed images will not change the detection of positive samples. On the other hand, supplementing with positive samples of the same class may lead to extra positive clues, which is unfair to the experiment.

**eAppendix 3. Framework development**

**Composite diagnostic classification**. In this paper, we develop a CHD diagnostic model to comprehensively utilize multimodal data with various scanning views. Although each modality per view of TTE holds a probability of CHD prediction, we do not simply weigh these probabilities. Instead, we aggregate the embeddings from different views and modalities, which can be complementary to each other, to establish cross-view and cross-modal features so that automatically generate more accurate and robust inferences.

**eAppendix 4. Training and testing settings**

All models are trained from scratch on our CHD dataset rather than fine-tuned with the pre-trained parameters on other datasets, such as ImageNet(2). We train the classification model for 100 epochs using the Adam optimizer with a learning rate of 1×10^-5^ and employ the batch size of 16. The dropout percentage for AlexNet is 50%. Cross entropy is adopted as the loss function in our work.

All models are performed in Python with Pytorch (https://pytorch.org/) platform based on a GeForce RTX® 3090 GPU. Training time for the view classification model with a training ratio of 80% is about 1.25h. For the multi-model model with five scanning views, it takes about 0.52-1.11h to train the model based on the training ratio ranging from 40% to 80%. When the views or modalities adopted by the model decrease, the parameters of the model will decrease, and the required training time will reduce accordingly. The inference time can be lower than 2.3ms for CHD classification per subject.

**eAppendix 5. Statistical Analysis**

For the normal/patient classification, the positive and negative correspond to the CHD and healthy control, respectively. In addition, we calculate the widely accepted metrics of accuracy (ACC), precision, sensitivity (i.e., recall), and specificity, which can be formulated as:

$$\begin{aligned} ACC=\frac{TP+TN}{TP+TN+FP+FN},\#\left( 1 \right) \end{aligned}$$

$$\begin{aligned} precision=\frac{TP}{TP+FP},\#\left( 2 \right) \end{aligned}$$

$$\begin{aligned} sensitivity=\frac{TP}{TP+FN},\#\left( 3 \right) \end{aligned}$$

$$\begin{aligned} specificity=\frac{TN}{TN+FP},\#\left( 4 \right) \end{aligned}$$

where TP, TN, FP, and FN are the true positive, true negative, false positive, and false negative, respectively. For the three-class classification of negative/ASD/VSD, we adopt the metrics of mean average precision (mAP) and mean intersection over union (mIoU):

$$\begin{aligned} mAP=\frac{\sum_{i=1}^{C} {precision}_{i}}{C},\#\left( 5 \right) \end{aligned}$$

$$\begin{aligned} IoU=\frac{TP}{TP+FP+FN},\#\left( 6 \right) \end{aligned}$$

$$\begin{aligned} mIoU=\frac{\sum_{i=1}^{C} {IoU}_{i}}{C},\#\left( 7 \right) \end{aligned}$$

where TP, TN, FP, and FN are the true positive, true negative, false positive, and false negative samples for each class. It can be seen that we first calculate the precision and IoU value for each class and then average per metric according to the total number of classes $C$ ($C=3$ in our dataset) to obtain the final mAP and mIoU values, respectively.

**eAppendix 6. Structure of DL models**

Deep learning, with the powerful ability to exploit morphological and textural patterns in images, enables extracting informative representations in a self-taught manner, thereby offloading the burden of feature engineering from experts in conventional computer-aided diagnostic systems and enabling data-driven latent feature exploration. The scanning view classification model is based on the ResNet18(3) architecture and CHD diagnostic classification models are built with four types of DL structures, ResNet18, AlexNet(4), VggNet(5), and ResNet50(3). These four networks are all Convolutional Neural Networks (CNNs), which have been shown to have powerful image processing and feature learning capabilities in many image tasks. Compared with ResNet18, AlexNet, and VggNet have completely different network structures and processing steps. Although ResNet50 and ResNet18 belong to the ResNet series of networks with residual connections but have entirely different "bottleneck" architecture and network depths. AlexNet utilizes relatively large kernels in convolution layers to learn image patterns and adopts a dropout scheme to reduce model overfitting. Vgg13 follows the dropout scheme but dedicates to obtaining a larger image receptive field by stacking multiple small filters in the convolution layers to construct a deeper network. The structures of these four networks are shown in eFigure 2. Instead of employing the most state-of-the-art models, which are always complex and hard to train, we choose these four typical and widely used networks so that we can better validate whether DL models can automate the analysis of the multi-modal transthoracic echocardiograms (TTE) from different scanning views for the detection of CHD.

**eAppendix 7. Diagnostic stability with more DL networks**

To explore the stability of the proposed diagnostic framework, we further employed more deep neural networks, AlexNet(4) and Vgg13(5), and ResNet50(3) (similar to ResNet18 but with a deeper structure), to conduct diagnostic classification. With a training data percentage of 80%, the five-view multi-modal model based on the AlexNet, Vgg13, and ResNet50 structures achieved a binary classification accuracy and AUC of more than 0.977 and 0.992, while a three-class classification accuracy of more than 0.971. ResNet18, AlexNet, Vgg13, and ResNet50 all achieved good CHD detection results, which further proved the effectiveness of our diagnostic framework based on the DL techniques.

**eAppendix 8. High-risk region visualziation**

In recognition of 2D TTEs, for ASDs, in the SXLAX view, our model paid attention to the enlarged right atrium and the defect of the atrial septum; in the PSLAX view, our model concentrated on the right ventricle with an enlarged inner diameter and the left atrium with reduced inner diameter; in the PSSAX view, the pulmonary artery with enlarged inner diameter attracted more attention; interestingly, while the sampled ASDs showed no abnormality in SSLAX view, and our model showed no ROI. For VSDs, in the A4C view, our model focused on the defect of the ventricular septum, the left ventricle with an enlarged inner diameter; in the SXLAX view, the enlarged left atrium was recognized by our model; in the PSLAX view, our model concentrated on the defect of the ventricular septum, left ventricle and left atrium with enlarged inner diameter; in PSSAX view, the pulmonary artery with an enlarged inner diameter was weighted more by our model; similarly, the example VSDs presented no abnormal in SSLAX view, while our model showed no ROI.

In recognition of Doppler TTEs, compared with the above-mentioned 2D TTEs, it is more inclined to recognize abnormal blood flow signals and chambers with reduced inner diameters by CAM analysis. For ASDs, in the A4C view, our model paid more attention to the atrial septum and left atrium, corresponding to the defect of the atrial septum, the abnormal left-to-right shunt signal at the defect, and the left atrium with reduced inner diameter; in the SXLAX view, our model focused on the abnormal left-to-right shunt signal of the defect, left atrium with reduced inner diameter; in the PSLAX view, our model focused on the left atrium with decreased inner diameter; in the PSSAX view, our model concentrated on the slightly increased pulmonary artery blood flow signal; the example ASD images showed no abnormality in the SSLAX section, and our model correspondingly generated no ROI. For VSDs, in the A4C view, the ventricular septum and left ventricle attracted more attention, which corresponds to the defect of the ventricular septum, the abnormal left-to-right shunt signal at the defect, and the left ventricle with increased inner diameter; in PSSAX view, our model focused on the slightly growing pulmonary artery blood flow signal; in SXLAX view, our model focused on the enlarged inner diameter of left atrium; the example SSLAX images showed no abnormality, and our model showed no ROI.

**References**

1. Wang J, Liu X, Wang F et al. Automated interpretation of congenital heart disease from multi-view echocardiograms. Medical image analysis 2021;69:101942.

2. Deng J, Dong W, Socher R, Li L-J, Li K, Fei-Fei L. Imagenet: A large-scale hierarchical image database. 2009 IEEE conference on computer vision and pattern recognition: IEEE, 2009:248-255.

3. He K, Zhang X, Ren S, Sun J. Deep residual learning for image recognition. Proceedings of the IEEE conference on computer vision and pattern recognition, 2016:770-778.

4. Krizhevsky A, Sutskever I, Hinton GE. ImageNet classification with deep convolutional neural networks. Communications of the ACM 2017;60:84-90.

5. Simonyan K, Zisserman AJapa. Very deep convolutional networks for large-scale image recognition. arXiv preprint arXiv:14091556 2014.

**eFigure 1. The confusion matrix of view classification with the training data ratio of 80% for the within-center evaluation.**


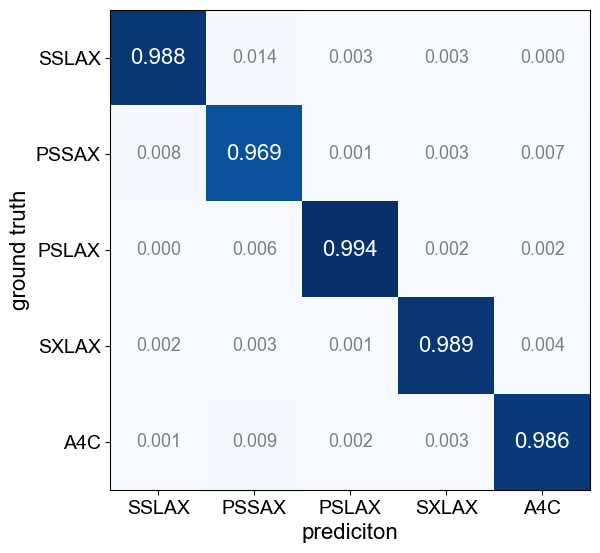


**eFigure 2. The DL network structures adopted in our framework.**


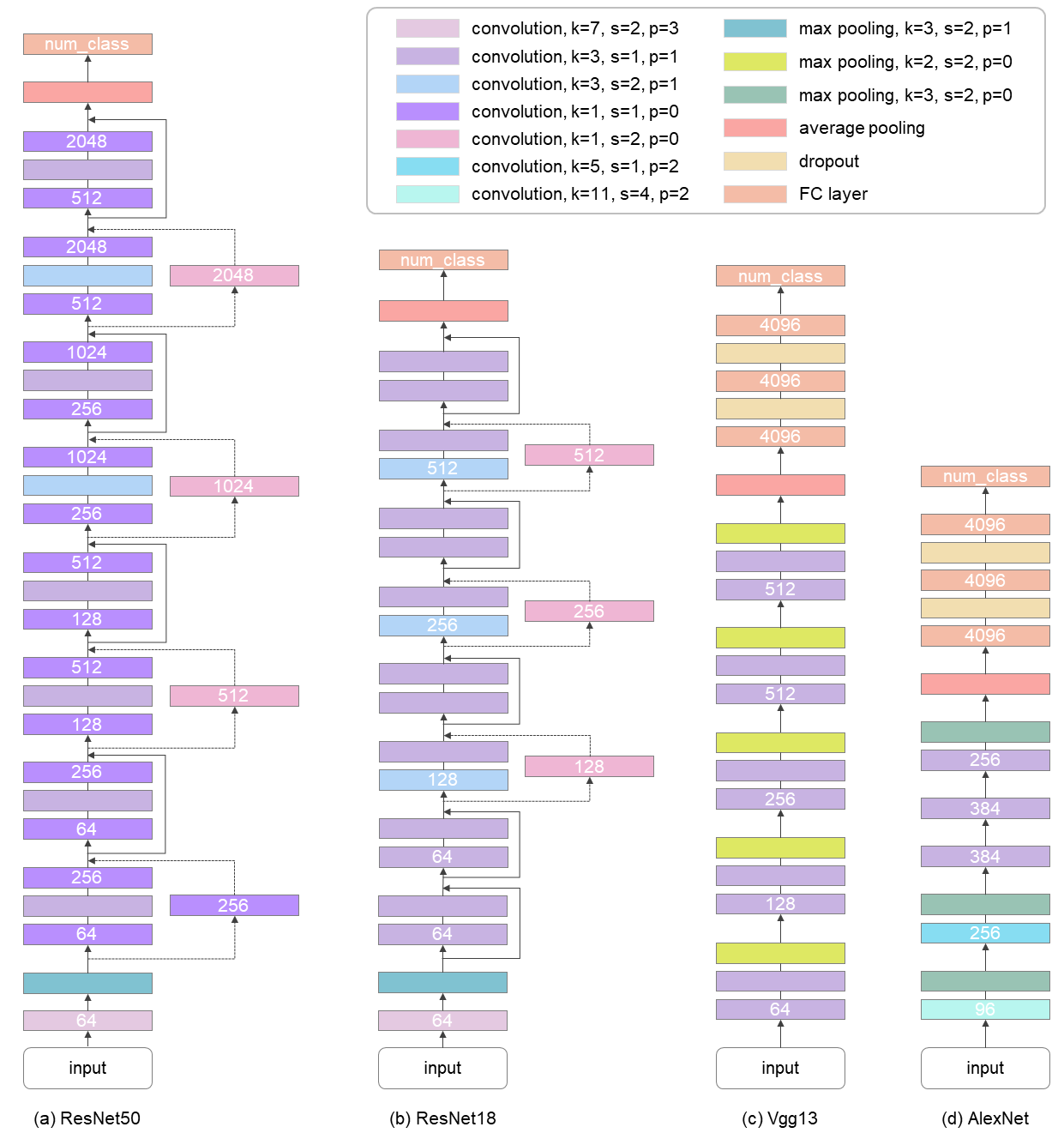


**eFigure 3. Testing images with corresponding CAMs.**


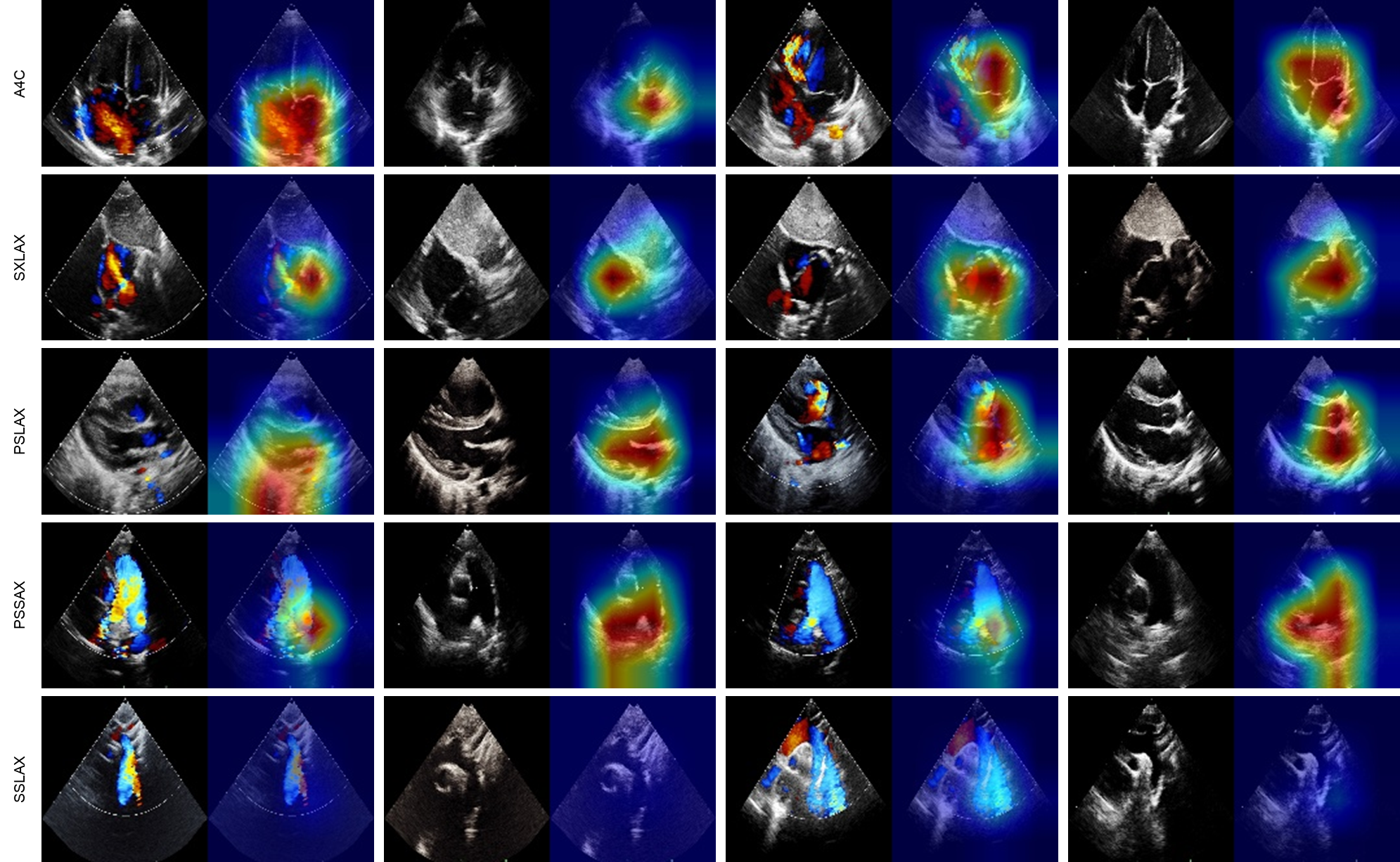


The left two columns indicate ASD, and the right two columns signify VSD. The red area shows the location to which the model pays the most attention.

**eFigure 4. Feature observation of the DL network.**


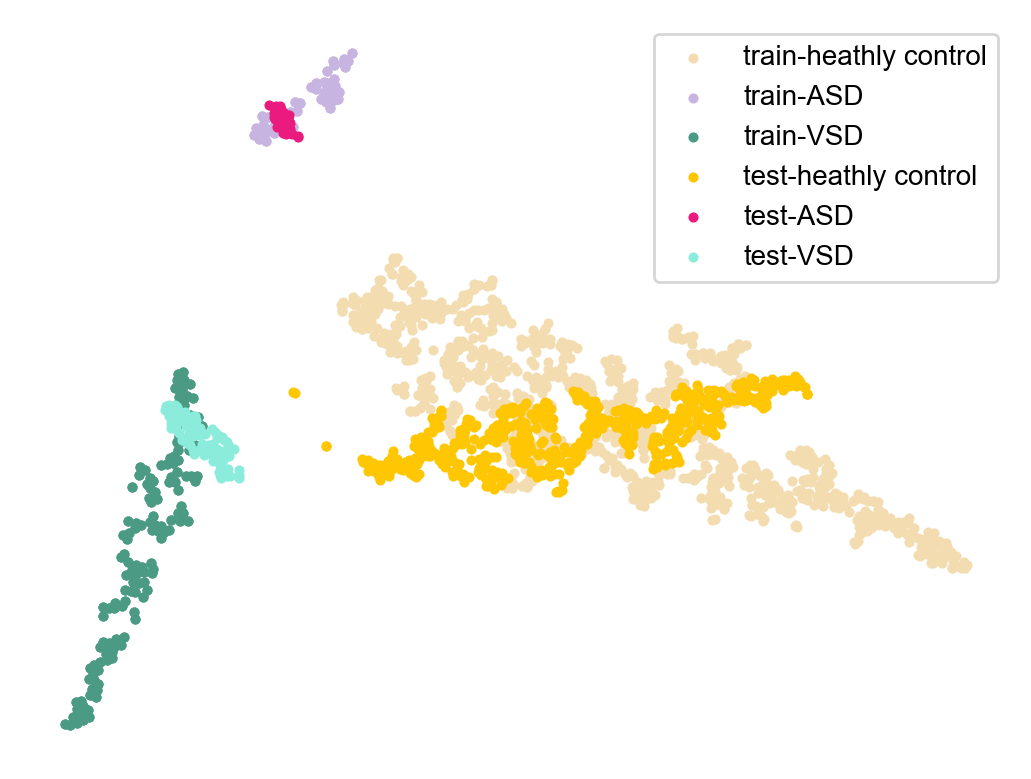


Two-dimensional (2D) t-distributed stochastic neighbor embedding (tSNE) of activations from the penultimate hidden layer of ResNet18 for the first dataset. Individual points correspond to representations of various subjects during training and testing (training set percentage=60%).

**eFigure 5. Keyframe selection flowchart.**

**
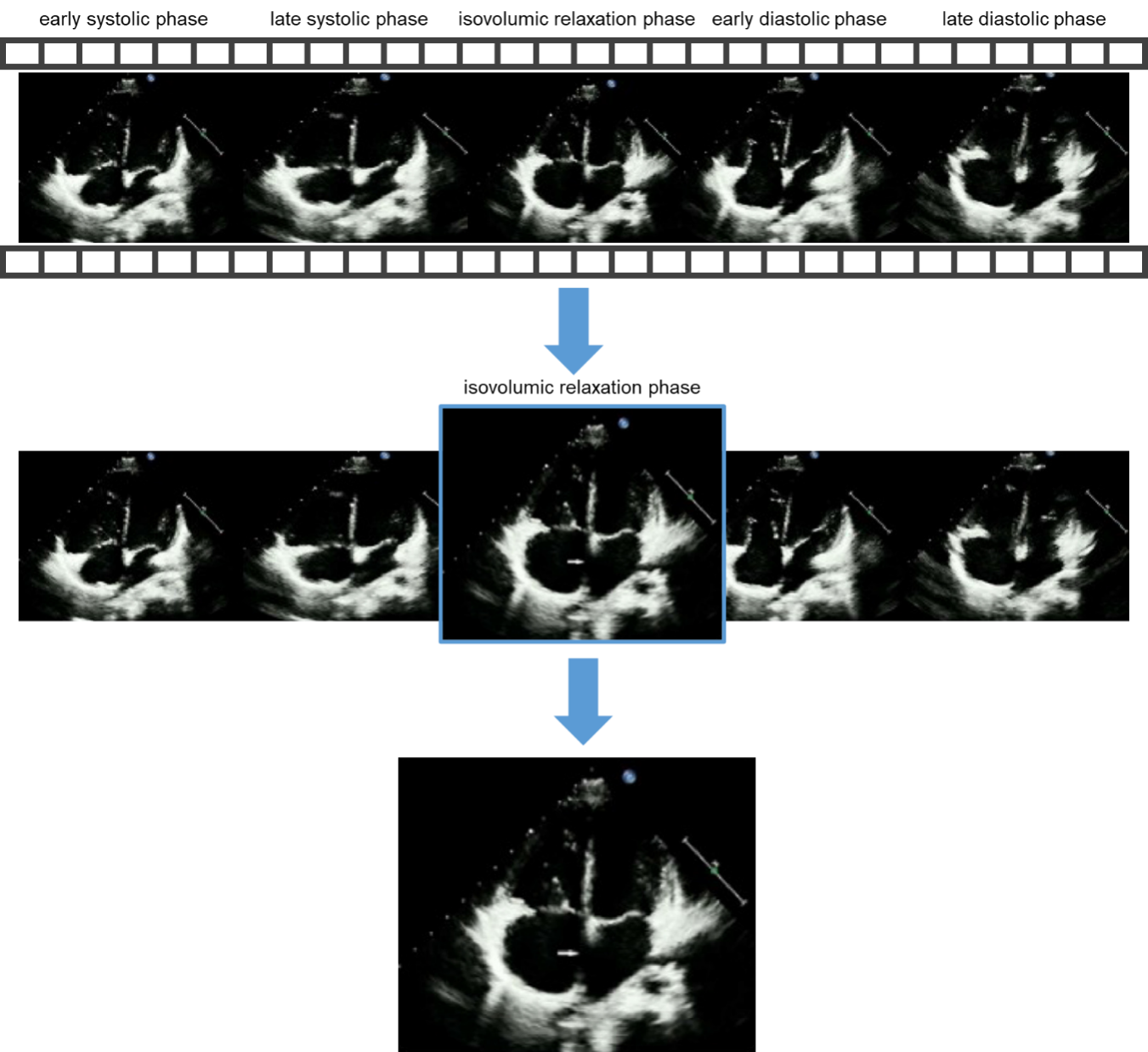
**

The flowchart of keyframe selection from the A4C TTE video of an ASD patient.

**eTable 1. The CHD diagnostic results based on automatic view identification.**

(a) Within-center evaluation

| **Variable** | | **Training proportion of 80%** | | |  | **Training proportion of 60%** | | |
| --- | --- | --- | --- | --- | --- | --- | --- | --- |
|  |  | **Bi-ACC** | **Bi-AUC** | **3-ACC** |  | **Bi-ACC** | **Bi-AUC** | **3-ACC** |
| Three-views | | | | | | | | |
|  | 2D | 0.958±0.002 | 0.966±0.002 | 0.940±0.001 |  | 0.931±0.002 | 0.939±0.002 | 0.921±0.002 |
|  | Doppler | 0.963±0.003 | 0.979±0.002 | 0.952±0.002 |  | 0.946±0.002 | 0.948±0.002 | 0.925±0.002 |
|  | Multi-modal | 0.979±0.002 | 0.987±0.002 | 0.969±0.002 |  | 0.960±0.001 | 0.974±0.002 | 0.946±0.001 |
| Five-views | | | | | | | | |
|  | 2D | 0.973±0.001 | 0.964±0.002 | 0.963±0.002 |  | 0.953±0.002 | 0.971±0.002 | 0.944±0.002 |
|  | Doppler | 0.982±0.002 | 0.975±0.001 | 0.973±0.002 |  | 0.956±0.002 | 0.969±0.003 | 0.957±0.002 |
|  | Multi-modal | **0.988±0.001** | **0.992±0.001** | **0.984±0.001** |  | 0.975±0.002 | 0.987±0.002 | 0.973±0.003 |

(b) Cross-center evaluation

| **Variable** | | **Bi-ACC** | **Bi-AUC** | **3-ACC** |  |
| --- | --- | --- | --- | --- | --- |
| Three-views | | | | |  |
|  | 2D | 0.942±0.003 | 0.966±0.001 | 0.943±0.001 |  |
|  | Doppler | 0.968±0.003 | 0.969±0.001 | 0.946±0.001 |  |
|  | Multi-modal | 0.974±0.003 | 0.980±0.004 | 0.963±0.001 |  |
| Five-views | | | | |  |
|  | 2D | 0.966±0.002 | 0.958±0.003 | 0.959±0.001 |  |
|  | Doppler | 0.983±0.001 | 0.977±0.002 | 0.969±0.002 |  |
|  | Multi-modal | **0.984±0.001** | **0.988±0.002** | **0.981±0.001** |  |

"Bi-ACC" and "Bi-AUC" mean the accuracy and AUC values of the binary classification (negative/positive), respectively, and "3-ACC" indicates the accuracy of three-class classification (negative/ASD/VSD). "Three view" denotes the fusion of A4C, SXLAX, and PSLAX and "Five view" denotes the fusion of all five scanning views. Based on the prediction results of the view classification model (with an 8:2 ratio for training and testing in the first dataset), we further perform the CHD detection based on the diagnostic classification model trained with proportions of 80% and 60%, respectively. Based on the CHD classification model trained with 80% of the first dataset, the cross-center test is further conducted.

**eTable 2. CHD recognition results of our multi-modal CHD identification model with various scanning views.**

(a) Within-center evaluation

| **Variable** | | | **Training proportion of 80%** | | |  | **Training proportion of 70%** | | |  | **Training proportion of 60%** | | |
| --- | --- | --- | --- | --- | --- | --- | --- | --- | --- | --- | --- | --- | --- |
|  |  |  | **Bi-ACC** | **Bi-AUC** | **3-ACC** |  | **Bi-ACC** | **Bi-AUC** | **3-ACC** |  | **Bi-ACC** | **Bi-AUC** | **3-ACC** |
| Single-view | | | | | | | | | | | | | |
|  | SSLAX | | | | | | | | | | | | |
|  |  | 2D | 0.831±0.003 | 0.721±0.002 | 0.819±0.002 |  | 0.848±0.003 | 0.750±0.002 | 0.836±0.002 |  | 0.816±0.005 | 0.717±0.003 | 0.811±0.002 |
|  |  | Doppler | 0.850±0.002 | 0.759±0.003 | 0.846±0.001 |  | 0.848±0.003 | 0.766±0.002 | 0.840±0.001 |  | 0.845±0.002 | 0.703±0.005 | 0.839±0.001 |
|  |  | Multi-modal | 0.873±0.004 | 0.818±0.004 | 0.864±0.002 |  | 0.869±0.002 | 0.828±0.001 | 0.851±0.004 |  | 0.857±0.003 | 0.789±0.004 | 0.847±0.000 |
|  | PSSAX | | | | | | | | | | | | |
|  |  | 2D | 0.794±0.002 | 0.724±0.004 | 0.767±0.003 |  | 0.778±0.002 | 0.716±0.003 | 0.767±0.001 |  | 0.774±0.002 | 0.713±0.005 | 0.766±0.001 |
|  |  | Doppler | 0.836±0.002 | 0.764±0.004 | 0.818±0.002 |  | 0.826±0.003 | 0.759±0.001 | 0.816±0.003 |  | 0.821±0.002 | 0.759±0.003 | 0.804±0.001 |
|  |  | Multi-modal | 0.868±0.001 | 0.847±0.003 | 0.865±0.002 |  | 0.865±0.001 | 0.829±0.002 | 0.832±0.002 |  | 0.848±0.002 | 0.824±0.003 | 0.837±0.001 |
|  | PSLAX | | | | | | | | | | | | |
|  |  | 2D | 0.805±0.004 | 0.731±0.002 | 0.783±0.002 |  | 0.780±0.002 | 0.726±0.001 | 0.765±0.003 |  | 0.770±0.002 | 0.728±0.002 | 0.760±0.002 |
|  |  | Doppler | 0.887±0.003 | 0.842±0.003 | 0.877±0.003 |  | 0.870±0.004 | 0.833±0.002 | 0.862±0.003 |  | 0.867±0.003 | 0.826±0.005 | 0.857±0.002 |
|  |  | Multi-modal | 0.913±0.004 | 0.898±0.003 | 0.898±0.006 |  | 0.904±0.001 | 0.889±0.002 | 0.897±0.001 |  | 0.896±0.002 | 0.862±0.003 | 0.891±0.002 |
|  | SXLAX | | | | | | | | | | | | |
|  |  | 2D | 0.829±0.004 | 0.765±0.003 | 0.817±0.002 |  | 0.825±0.003 | 0.761±0.002 | 0.811±0.001 |  | 0.811±0.001 | 0.750±0.004 | 0.794±0.002 |
|  |  | Doppler | 0.884±0.004 | 0.823±0.005 | 0.855±0.003 |  | 0.874±0.001 | 0.786±0.002 | 0.845±0.001 |  | 0.864±0.002 | 0.809±0.002 | 0.833±0.003 |
|  |  | Multi-modal | 0.924±0.002 | 0.915±0.003 | 0.876±0.005 |  | 0.890±0.002 | 0.849±0.002 | 0.868±0.002 |  | 0.864±0.002 | 0.861±0.003 | 0.845±0.002 |
|  | A4C | | | | | | | | | | | | |
|  |  | 2D | 0.809±0.003 | 0.798±0.003 | 0.776±0.003 |  | 0.791±0.003 | 0.790±0.003 | 0.766±0.002 |  | 0.764±0.002 | 0.771±0.004 | 0.757±0.002 |
|  |  | Doppler | 0.858±0.004 | 0.850±0.003 | 0.831±0.002 |  | 0.838±0.002 | 0.842±0.003 | 0.822±0.002 |  | 0.833±0.002 | 0.827±0.002 | 0.813±0.001 |
|  |  | Multi-modal | 0.918±0.001 | 0.905±0.002 | 0.913±0.003 |  | 0.905±0.004 | 0.883±0.002 | 0.893±0.005 |  | 0.900±0.001 | 0.867±0.002 | 0.888±0.002 |

| **Variable** | | | **Training proportion of 80%** | | |  | **Training proportion of 70%** | | |  | **Training proportion of 60%** | | |
| --- | --- | --- | --- | --- | --- | --- | --- | --- | --- | --- | --- | --- | --- |
|  |  |  | **Bi-ACC** | **Bi-AUC** | **3-ACC** |  | **Bi-ACC** | **Bi-AUC** | **3-ACC** |  | **Bi-ACC** | **Bi-AUC** | **3-ACC** |
| Three-views | | | | | | | | | | | | | |
|  |  | 2D | 0.971±0.002 | 0.987±0.001 | 0.954±0.002 |  | 0.957±0.002 | 0.975±0.001 | 0.951±0.002 |  | 0.957±0.002 | 0.975±0.002 | 0.942±0.002 |
|  |  | Doppler | 0.982±0.002 | 0.990±0.000 | 0.971±0.003 |  | 0.972±0.002 | 0.969±0.001 | 0.968±0.002 |  | 0.960±0.001 | 0.969±0.001 | 0.938±0.004 |
|  |  | Multi-modal | 0.985±0.001 | 0.994±0.001 | 0.964±0.002 |  | 0.975±0.001 | 0.988±0.001 | 0.969±0.002 |  | 0.972±0.002 | 0.982±0.000 | 0.956±0.003 |
| Five-views | | | | | | | | | | | | | |
|  |  | 2D | 0.980±0.001 | 0.994±0.001 | 0.974±0.002 |  | 0.977±0.001 | 0.984±0.001 | 0.973±0.002 |  | 0.965±0.002 | 0.981±0.001 | 0.959±0.002 |
|  |  | Doppler | 0.989±0.003 | 0.992±0.000 | 0.982±0.002 |  | 0.975±0.002 | 0.983±0.001 | 0.974±0.003 |  | 0.966±0.003 | 0.982±0.002 | 0.964±0.005 |
|  |  | Multi-modal | **0.994±0.002** | **0.996±0.000** | **0.991±0.002** |  | 0.990±0.003 | 0.991±0.001 | 0.984±0.002 |  | 0.988±0.001 | 0.987±0.002 | 0.981±0.001 |

(b) Cross-center evaluation

| **Variable** | | **Bi-ACC** | **Bi-AUC** | **3-ACC** |  |
| --- | --- | --- | --- | --- | --- |
| Three-views | | | | |  |
|  | 2D | 0.965±0.004 | 0.987±0.003 | 0.950±0.001 |  |
|  | Doppler | 0.979±0.001 | 0.988±0.001 | 0.964±0.001 |  |
|  | Multi-modal | 0.983±0.003 | 0.986±0.002 | 0.960±0.003 |  |
| Five-views | | | | |  |
|  | 2D | 0.975±0.001 | 0.985±0.003 | 0.972±0.003 |  |
|  | Doppler | 0.986±0.004 | 0.987±0.002 | 0.978±0.001 |  |
|  | Multi-modal | **0.990±0.003** | **0.993±0.001** | **0.986±0.001** |  |

"Bi-ACC" and "Bi-AUC" mean the accuracy and AUC values of the binary classification (normal/patient classifier), respectively, and "3-ACC" indicates the accuracy of three-class classification (negative/ASD/VSD). "Three views" indicates the fusion of A4C, PSLAX, and SXLAX and "Five views" denotes the fusion of five scanning views. Based on the CHD classification model trained with 80% of the first dataset, the cross-center evaluation is further conducted.

**eTable 3. Quantitative diagnostic results based on different modalities and scanning views with 50% and 40% training ratios for within-center evaluation.**

| **Variable** | | **Training proportion of 50%** | | |  | **Training proportion of 40%** | | |
| --- | --- | --- | --- | --- | --- | --- | --- | --- |
|  |  | **Bi-ACC** | **Bi-AUC** | **3-ACC** |  | **Bi-ACC** | **Bi-AUC** | **3-ACC** |
| Three-views | | | | | | | | |
|  | 2D | 0.932±0.002 | 0.932±0.002 | 0.932±0.002 |  | 0.918±0.001 | 0.918±0.001 | 0.918±0.001 |
|  | Doppler | 0.938±0.002 | 0.938±0.002 | 0.938±0.002 |  | 0.937±0.002 | 0.937±0.002 | 0.937±0.002 |
|  | Multi-modal | 0.966±0.001 | 0.966±0.001 | 0.966±0.001 |  | 0.961±0.001 | 0.961±0.001 | 0.961±0.001 |
| Five-views | | | | | | | | |
|  | 2D | 0.947±0.002 | 0.947±0.002 | 0.947±0.002 |  | 0.944±0.002 | 0.944±0.002 | 0.944±0.002 |
|  | Doppler | 0.952±0.003 | 0.952±0.003 | 0.952±0.003 |  | 0.950±0.004 | 0.950±0.004 | 0.950±0.004 |
|  | Multi-modal | 0.977±0.001 | 0.977±0.001 | 0.977±0.001 |  | 0.973±0.002 | 0.973±0.002 | 0.973±0.002 |

"Bi-ACC" and "Bi-AUC" mean the accuracy and AUC values of the binary classification (normal/patient classifier), respectively, and "3-ACC" indicates the accuracy of three-class classification (negative/ASD/VSD). "Three views" indicates the fusion of A4C, PSLAX, and SXLAX and "Five views" denotes the fusion of five scanning views.

**eTable 4. Results of diagnostic model based on different DL networks on the within-center test set.**

| **Variable** | | | **Training proportion of 80%** | | |  | **Training proportion of 60%** | | |
| --- | --- | --- | --- | --- | --- | --- | --- | --- | --- |
|  |  |  | **Bi-ACC** | **Bi-AUC** | **3-ACC** |  | **Bi-ACC** | **Bi-AUC** | **3-ACC** |
| AlexNet | | | | | | | | | |
|  | Three-views | | | | | | | | |
|  |  | 2D | 0.942±0.005 | 0.968±0.000 | 0.916±0.001 |  | 0.910±0.001 | 0.950±0.001 | 0.867±0.003 |
|  |  | Doppler | 0.960±0.001 | 0.991±0.000 | 0.925±0.001 |  | 0.928±0.001 | 0.964±0.000 | 0.901±0.001 |
|  |  | Multi-modal | 0.967±0.001 | 0.994±0.000 | 0.955±0.001 |  | 0.952±0.001 | 0.966±0.000 | 0.938±0.001 |
|  | Five-views | | | | | | | | |
|  |  | 2D | 0.940±0.002 | 0.976±0.000 | 0.921±0.002 |  | 0.939±0.001 | 0.957±0.000 | 0.911±0.001 |
|  |  | Doppler | 0.974±0.001 | 0.989±0.000 | 0.961±0.001 |  | 0.952±0.002 | 0.968±0.000 | 0.942±0.001 |
|  |  | Multi-modal | 0.977±0.000 | 0.996±0.000 | 0.971±0.001 |  | 0.969±0.001 | 0.973±0.000 | 0.950±0.001 |
| Vgg13 | | | | | | | | | |
|  | Three-views | | | | | | | | |
|  |  | 2D | 0.947±0.002 | 0.971±0.002 | 0.942±0.002 |  | 0.926±0.001 | 0.960±0.001 | 0.918±0.001 |
|  |  | Doppler | 0.963±0.001 | 0.989±0.002 | 0.952±0.002 |  | 0.931±0.001 | 0.971±0.003 | 0.925±0.001 |
|  |  | Multi-modal | 0.977±0.001 | 0.990±0.002 | 0.971±0.000 |  | 0.950±0.001 | 0.976±0.002 | 0.950±0.001 |
|  | Five-views | | | | | | | | |
|  |  | 2D | 0.948±0.001 | 0.978±0.001 | 0.937±0.002 |  | 0.945±0.001 | 0.975±0.001 | 0.936±0.001 |
|  |  | Doppler | 0.968±0.002 | 0.987±0.001 | 0.958±0.001 |  | 0.954±0.001 | 0.981±0.003 | 0.950±0.001 |
|  |  | Multi-modal | 0.977±0.001 | 0.992±0.001 | 0.974±0.000 |  | 0.975±0.001 | 0.987±0.002 | 0.974±0.001 |

| **Variable** | | | **Training proportion of 80%** | | |  | **Training proportion of 60%** | | |
| --- | --- | --- | --- | --- | --- | --- | --- | --- | --- |
|  |  |  | **Bi-ACC** | **Bi-AUC** | **3-ACC** |  | **Bi-ACC** | **Bi-AUC** | **3-ACC** |
| ResNet50 | | | | | | | | | |
|  | Three-views | | | | | | | | |
|  |  | 2D | 0.945±0.002 | 0.978±0.002 | 0.938±0.004 |  | 0.903±0.004 | 0.945±0.002 | 0.880±0.006 |
|  |  | Doppler | 0.948±0.006 | 0.986±0.001 | 0.939±0.002 |  | 0.903±0.005 | 0.944±0.003 | 0.900±0.003 |
|  |  | Multi-modal | 0.980±0.002 | 0.998±0.001 | 0.968±0.004 |  | 0.959±0.003 | 0.987±0.001 | 0.950±0.003 |
|  | Five-views | | | | | | | | |
|  |  | 2D | 0.945±0.004 | 0.980±0.002 | 0.940±0.007 |  | 0.918±0.005 | 0.955±0.003 | 0.922±0.004 |
|  |  | Doppler | 0.960±0.005 | 0.984±0.002 | 0.948±0.004 |  | 0.958±0.007 | 0.960±0.004 | 0.951±0.007 |
|  |  | Multi-modal | 0.985±0.002 | 0.999±0.000 | 0.977±0.001 |  | 0.980±0.003 | 0.993±0.001 | 0.962±0.003 |

"Bi-ACC" and "Bi-AUC" mean the accuracy and AUC values of the binary classification (normal/patient classifier), respectively, and "3-ACC" indicates the accuracy of three-class classification (negative/ASD/VSD). "Three views" indicates the fusion of A4C, PSLAX, and SXLAX and "Five views" denotes the fusion of five scanning views.
